# The effects of positive end-expiratory pressure on cardiac function: a comparative echocardiography-conductance catheter study

**DOI:** 10.1007/s00392-022-02014-1

**Published:** 2022-04-06

**Authors:** David Berger, Olivier Wigger, Stefano de Marchi, Martin R. Grübler, Andreas Bloch, Reto Kurmann, Odile Stalder, Kaspar Felix Bachmann, Stefan Bloechlinger

**Affiliations:** 1 Department of Intensive Care Medicine, Inselspital, Bern University Hospital, University of Bern, 3010 Bern, Switzerland; 2 Department of Cardiology, Inselspital, Bern University Hospital, University of Bern, Bern, Switzerland; 3 Klinik Für Kardiologie, Kantonsspital Winterthur, Winterthur, Switzerland; 4 Zentrum Für Intensivmedizin, Kantonsspital Luzern, Luzern, Switzerland; 5 Klinik Für Kardiologie, Kantonsspital Luzern, Luzern, Switzerland; 6 CTU Bern, University of Bern, Bern, Switzerland; 7 Department of Anesthesiology and Pain Medicine, Inselspital, Bern University Hospital,, University of Bern, Bern, Switzerland

**Keywords:** Diastolic function, Echocardiography, End-diastolic pressure, Volume relationship, End-systolic pressure, Volume relationship, Positive end-expiratory pressure, Mechanical ventilation

## Abstract

**Background:**

Echocardiographic parameters of diastolic function depend on cardiac loading conditions, which are altered by positive pressure ventilation. The direct effects of positive end-expiratory pressure (PEEP) on cardiac diastolic function are unknown.

**Methods:**

Twenty-five patients without apparent diastolic dysfunction undergoing coronary angiography were ventilated noninvasively at PEEPs of 0, 5, and 10 cmH_2_O (in randomized order). Echocardiographic diastolic assessment and pressure–volume-loop analysis from conductance catheters were compared. The time constant for pressure decay (*τ*) was modeled with exponential decay. End-diastolic and end-systolic pressure volume relationships (EDPVRs and ESPVRs, respectively) from temporary caval occlusion were analyzed with generalized linear mixed-effects and linear mixed models. Transmural pressures were calculated using esophageal balloons.

**Results:**

*τ* values for intracavitary cardiac pressure increased with the PEEP (*n* = 25; no PEEP, 44 ± 5 ms; 5 cmH_2_O PEEP, 46 ± 6 ms; 10 cmH_2_O PEEP, 45 ± 6 ms; *p* < 0.001). This increase disappeared when corrected for transmural pressure and diastole length. The transmural EDPVR was unaffected by PEEP. The ESPVR increased slightly with PEEP. Echocardiographic mitral inflow parameters and tissue Doppler values decreased with PEEP [peak E wave (*n* = 25): no PEEP, 0.76 ± 0.13 m/s; 5 cmH_2_O PEEP, 0.74 ± 0.14 m/s; 10 cmH_2_O PEEP, 0.68 ± 0.13 m/s; *p* = 0.016; peak A wave (*n* = 24): no PEEP, 0.74 ± 0.12 m/s; 5 cmH_2_O PEEP, 0.7 ± 0.11 m/s; 10 cmH_2_O PEEP, 0.67 ± 0.15 m/s; *p* = 0.014; E’ septal (*n* = 24): no PEEP, 0.085 ± 0.016 m/s; 5 cmH_2_O PEEP, 0.08 ± 0.013 m/s; 10 cmH_2_O PEEP, 0.075 ± 0.012 m/s; *p* = 0.002].

**Conclusions:**

PEEP does not affect active diastolic relaxation or passive ventricular filling properties. Dynamic echocardiographic filling parameters may reflect changing loading conditions rather than intrinsic diastolic function. PEEP may have slight positive inotropic effects.

**Clinical trial registration:**

https://clinicaltrials.gov/ct2/show/NCT02267291, registered 17. October 2014.

**Graphical abstract:**

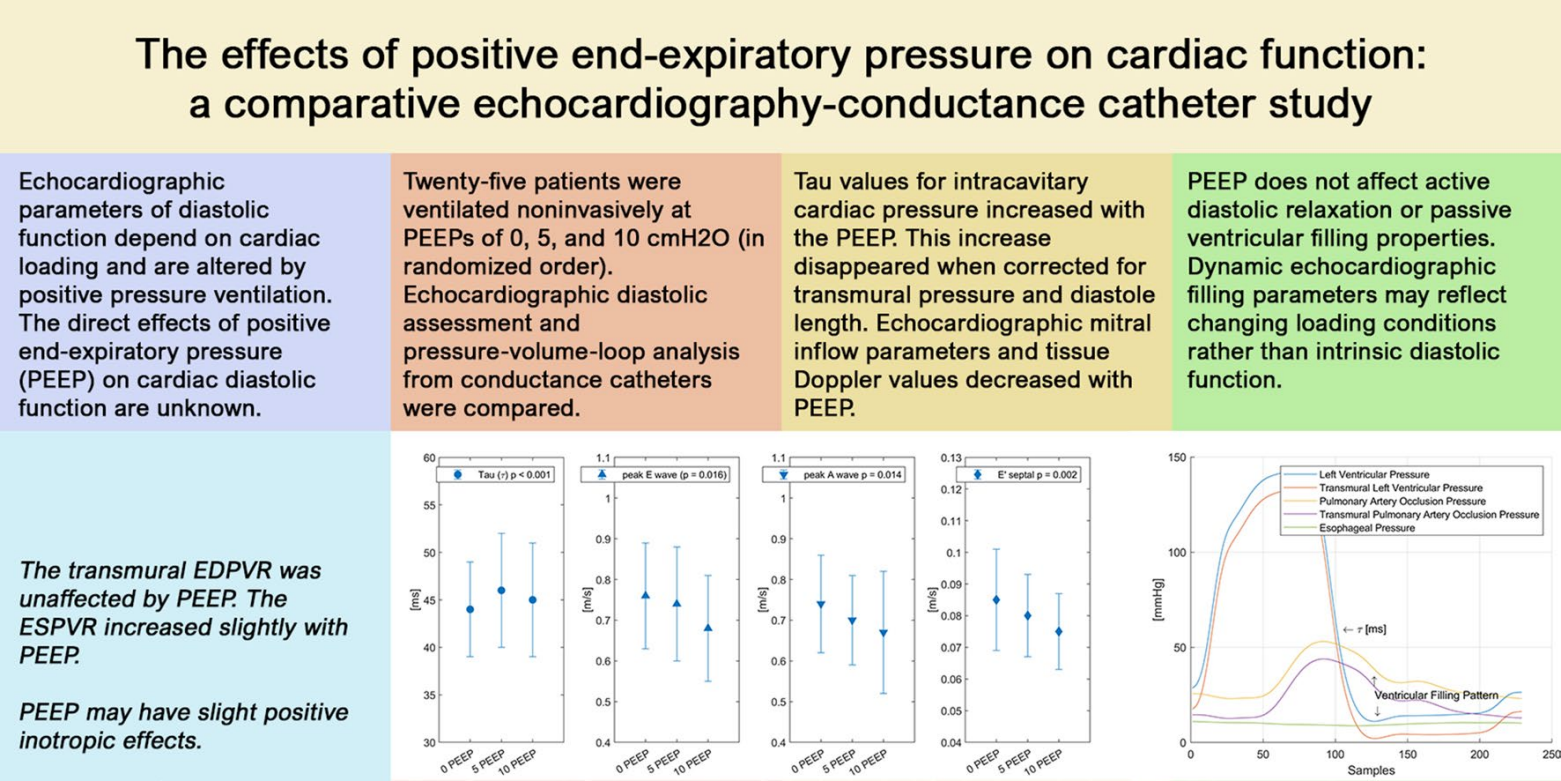

**Supplementary Information:**

The online version contains supplementary material available at 10.1007/s00392-022-02014-1.

## Background

Echocardiography is used widely to investigate cardiac function in patients on mechanical ventilation due to acute respiratory failure, cardiogenic pulmonary edema, or other critical illnesses, or for general anesthesia [1]. The echocardiographic parameters best suited for the evaluation of left or right ventricular dysfunction remain unclear. This issue has been given high priority in research agendas for critical care echocardiography [1, 2]. Diastolic dysfunction (diagnosed by echocardiography with tissue Doppler and mitral flow pattern analyses) is a frequent cause of weaning failure [3–6], has been termed “understudied” [7], and is used to guide therapeutic decision making [5, 8]. Questions remain as to whether Doppler-derived parameters truly reflect intrinsic diastolic cardiac properties or represent preload- and afterload-dependent filling phenomena [9, 10]. The positive intrathoracic pressure applied by mechanical ventilation leads to an overall preload reduction and an increase in right ventricular and decrease in left ventricular afterload [11–14]. Positive end-expiratory pressure (PEEP) influences the echocardiographic assessment of diastolic function in anesthetized and critically ill patients [15–17], questioning the validity of echo-Doppler assessments under mechanical ventilation. For the investigation of diastolic dysfunction independently of cardiac loading conditions, invasive pressure–volume-loop analysis with conductance catheters is the gold standard. Invasive animal studies have provided conflicting results [18, 19], and we are not aware of any reporting of invasively measured data from humans under mechanical ventilation. As the pleural space is the effective working environment of the heart, its transmural pressure may be approximated by subtracting the esophageal pressure from the intracavitary pressure [11, 20, 21].

The widespread use of positive pressure ventilation in anesthesia and critical care and the load dependency of Doppler-derived parameters for diastolic function underscore the need for the validation of echocardiographic diastology parameters measured under mechanical ventilation. With this study, we aimed to elucidate intrinsic ventricular properties under PEEP and to obtain an assembled picture of diastolic left ventricular function together with Doppler-derived filling parameters. We hypothesized that mitral inflow and tissue Doppler parameters would reflect altered cardiac loading conditions attributable to PEEP, rather than changes in myocardial properties.

## Materials and methods

### Participants

This prospective, controlled single-center observational study conducted at the University Hospital Bern was approved by the Ethics Committee of the Canton of Bern (KEK 104/14). Patients provided written informed consent. The report follows the STROBE guidelines. Adult patients with no known heart, pulmonary, renal, or esophageal disease scheduled for elective coronary angiography between June 2015 and January 2018 were included prospectively, unless diastolic dysfunction > grade 1 was present on transthoracic echocardiography.

### Protocol

**Fig. 1:**
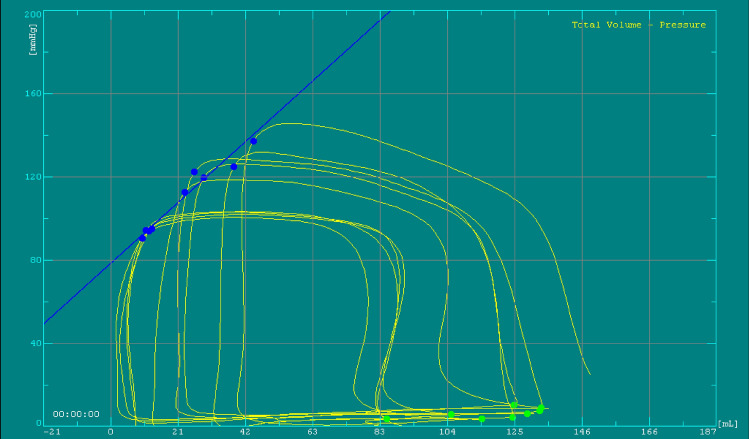
Exemplary loop family from a caval occlusion maneuver with the INCA device, recorded at a PEEP level of 10 cmH_2_O. The loop family corresponds to the echocardiography shown in Fig. 5

A pulmonary-artery balloon catheter was placed in the right pulmonary artery. Cardiac output was measured by thermodilution and the Fick method [22]. Left-ventricular pressure volume loops were obtained with a 7 French combined pressure-conductance catheter system (Sentron Europe BV, Roden, Netherlands and INCA; CDLeycom, Hengelo, Netherlands), calibrated as described previously [23]. An Amplatzer sizing balloon (Abbott Medical, The corporate village, Zaventeem, Belgium) was placed in the inferior vena cava for intermittent reduction of the preload (Fig. 1). Comprehensive transthoracic echocardiography (Vivid E9 and E95; GE Medical Systems, Glattbrugg, Switzerland) was performed during the pressure–volume-loop assessment. Data were stored for blinded offline analysis (EchoPac software, version 08; GE Medical Systems).

The assessment was performed with the participants breathing spontaneously, and with continuous positive airway pressure (CPAP) applied via a tight face mask; this mode of ventilation maintains continuous PEEP. Pressures of 5 and 10 cmH_2_O were applied in randomized order (Servo-i; Maquet, Solna, Sweden). An air-filled esophageal balloon (Nutrivent; Sidam s.r.l., Mirandola, Italy) [24, 25] was placed behind the left atrium under fluoroscopic guidance, with occlusion testing to confirm proper positioning [26, 27]. This balloon served as a surrogate for pleural pressure, which is needed for the calculation of transmural cardiac pressures. Pulmonary-artery and left-ventricular blood gases were taken at each stage of the assessment (spontaneous breathing, 5 cmH_2_O PEEP, and 10 cmH_2_O PEEP), together with noninvasive blood pressure, pulmonary artery pressure, and left-ventricular pressure–volume loops during normal respiration and expiratory holds in parallel with the echocardiographic assessment. At each stage, the cardiac preload was reduced temporarily by occlusion of the inferior vena cava to obtain pressure–volume loop families for end-systolic and end-diastolic pressure–volume relationships (ESPVR and EDPVR, respectively, Fig. 1). All invasive measurements were taken during expiratory hold to avoid additional pressure swings from respiration. Additional details are provided in Additional file 1.

### Data collection, processing, and analysis

Pressure data were entered into the dedicated acquisition system with standard pressure transducers, and subsequently extracted to Matlab (Release 2018b; MathWorks, Natick, Massachusetts, USA). The isovolumetric relaxation time constant (*τ*) was recalculated from heartbeats during expiratory hold using exponential (*τ*_Glantz_) and logistic (*τ*_logistic_) models for intraventricular and transmural ventricular pressures by subtraction of the esophageal pressure [28]. Diastole lengths were resampled to a standardized diastole length. For the intraventricular and transmural pressures, end-diastolic pressure–volume relationships were calculated using an exponential curve (*P* = *Ce*^*βV*^) fit to the diastolic pressure–volume points to determine the chamber stiffness constant (*β*) [29] and the pressure at an end-diastolic volume of 0 ml (= *C*). *V* equals chamber volume. Additional methodological details are provided in Additional file 1.

### Statistical analysis

The normality of the data distributions was assessed using the Shapiro–Wilk test and *Q*–*Q* plots. Comparisons among groups were made by one-way repeated-measures or Friedman’s analysis of variance, as appropriate, with post-hoc Bonferroni correction. ESPVRs were assessed using linear mixed-effects regression, with systolic pressure serving as the dependent variable. EDPVRs were assessed using generalized linear mixed models with a log link and gamma distribution for diastolic pressure. In all models, the volume, PEEP group, and interaction between them served as independent variables. Additionally, a random intercept and slope for volume were introduced at the participant level to allow for heterogeneity in the pressure–volume relationship among participants. For better interpretability, systolic and diastolic volumes were centered at their mean values. The analyses were performed with SPSS (version 21; IBM Corporation, Armonk, NY, USA) and Stata (version 16; StataCorp, College Station, TX, USA) software, and two-tailed *p* values < 0.05 were considered to be significant.

## Results

### Sample characteristics

30 cardiac patients (aged 18–60 years) consented to study participation. Five could not undergo assessment due to capacity constraints in the catheter laboratory. The analyses were performed with data from 25 patients (7 women, 18 men, Table 1). The sample for steady-state assessment comprised 1298 pressure–volume loops. After the exclusion of occlusion maneuver artifacts, 1598 and 1052 loops were entered into linear and generalized linear mixed-effects models for the assessment of ESPVRs and EDPVRs, respectively. The study procedures were performed without complication.

**Table 1:** Characteristics of the study population

Patients	*n*	25
Age	(years)	60.1 ± 6.3
Female gender	*n* (%)	7 (28)
Height	(cm)	170 ± 7
Weight	(kg)	78 ± 12
Body surface area	(m^2^)	1.9 ± 0.2
Oxygen consumption, calculated [22]	(ml/min)	236 ± 19
Co-morbidities		
Arterial hypertension	*n* (%)	16 (64)
Coronary heart disease	*n* (%)	8 (32)
Chronic renal insufficiency	*n* (%)	2 (8)
Diabetes mellitus	*n* (%)	4 (16)
COPD	*n* (%)	1 (4)
OSAS	*n* (%)	4 (16)
Medication		
Beta blocker	*n* (%)	5 (20)
ACE-Inhibitor / ATII-Antagonist	*n* (%)	14 (56)
Ca^2+^-Antagonist	*n* (%)	6 (24)
Inhalation therapy	*n* (%)	1(4)
Laboratory values		
Hemoglobin	(g/l)	133 ± 10
eGFR	ml/min	81 ± 17

Data are given as mean ± SD or *n* (%)

*COPD* chronic obstructive pulmonary disease, *OSAS* obstructive sleep apnea syndrome, *ACE* angiotensin-converting enzyme, *ATII* angiotensin II, *eGFR* estimated glomerular filtration rate (calculated using the KDIGO CKD-EPI formula)

The thermodilution cardiac output decreased slightly with increasing PEEP at stable stroke volumes due to a slower heart rate with higher PEEPs (Table 2). The pulmonary artery occlusion pressure and the esophageal pressure (as a surrogate of pleural pressure) increased with PEEP. Echocardiography showed a stable mean ejection fraction of 65% ± 5% with unchanging chamber volumes (Table 2).

**Table 2:** Baseline hemodynamic data

		*n*	Positive end-expiratory pressure			Friedman ANOVA
			0 cmH_2_O	5 cmH_2_O	10 cmH_2_O	*p* value
Hemodynamic data						
Non-invasive blood pressure						
Systolic	mmHg	24	125 (98–153)	130 (103–158)	127 (94–152)	0.384
Diastolic	mmHg	24	67 (50–88)	68 (55–108)	68 (46–81)	0.81
Mean	mmHg	24	88 (71–106)	86 (70–125)	86 (61–105)	0.587
Heart rate	min^−1^	25	68 (51–85) ^†^	65 (50–89)	63 (49–85)	**0.002**
Cardiac output						
Thermodilution	l/min	25	6.5 (3.6–9.0)^*,†^	5.8 (3.7–8.0)	5.8 (3.5–8.0)	**0.006**
Fick principle	l/min	25	5.4 (3.7–7.4)	5.4 (3.1–7.7)	5.2 (3.8–7.6)	0.069
Esophageal pressure	mmHg	25	7.4 (1.6–34)^*,†^	9.1 (3.8–62)^‡^	10.2 (1.25–20.9)	** < 0.001**
Mean pulmonary artery pressure	mmHg	24	17.8 (11.1–42.1)	19.2 (13.2–34.9)	18.3 (13.8–32.5)	0.687
Mean pulmonary artery occlusion pressure	mmHg	24	9.4 (5.6–15.4)^*,†^	11.6 (7.9–16.6)	11.9 (6.2–18.8)	**0.005**
Pulmonary vascular resistance	WU	25	1.36 ± 0.64	1.33 ± 0.51	1.36 ± 0.51	0.933^§^
Left ventricle						
EDV biplane	ml	18	109 (64–142)	117 (69–148)	115 (61–166)	0.157
ESV biplane	ml	18	36 (22–64)	37 (29–63)	36 (24–82)	0.799
EF Simpson	%	18	65 (55–75)	67 (50–74)	64 (51–71)	0.498
SV biplane	ml	18	75 (42–92)	75 (34–99)	72 (37–85)	0.101
Right ventricle						
S' tricuspid valve	m/s	19	0.12 (0.09–0.21)	0.11 (0.08–0.23)	0.12 (0.08–0.17)	0.416
TAPSE	cm	25	2.46(1.93– 3.26)^*,†^	2.3(1.93–3.0)^‡^	2.13 (1.66–3.0)	** < 0.001**
Blood oxygen saturation						
Left ventricular, SaO_2_	%	25	95 (90–98)^*,†^	96 (92–99)	96 (92–100)	***0.008***
Mixed venous, SmvO_2_	%	25	71.5 (63–78)	71 (64–98)	72 (64–79)	0.648

*p* < 0.05, post-hoc Wilcoxon test: *0 vs. 5 cmH_2_O, ^†^0 vs. 10 cmH_2_O, ^‡^5 vs. 10 cmH_2_O PEEP. ^§^Normal distribution, one-way repeated-measures ANOVA

*ANOVA* analysis of variance, *WU* Wood units, *EDV* end-diastolic volume, *ESV* end-systolic volume, *EF* ejection fraction, *SV* systolic volume, *TAPSE* tricuspid annular plane systolic excursion

*p*-values ≤0.05 were considered statistically significant and are indicated bold

### Invasive assessment and modeling properties

#### Systolic function and ventriculo-aortic coupling

The ejection fraction, end-systolic pressure, the pressure change over time (d*P*/d*t*_max,_ reflecting passive filling) and ESPVR slope (reflecting end-systolic elastance) did not alter with the PEEP. The aortic elastance did not change with increasing PEEP. The ratio of end-systolic to aortic elastance, an indicator of ventriculo-aortic coupling, remained unchanged with changes in PEEP, as did the stroke work and preload recruitable stroke work (Table 3). The linear mixed model revealed small, but significant, changes in the ESVPR and transmural ESPVR slopes (except for the transmural ESPVR at 5 cmH_2_O PEEP; Table 4). The changes in the intercepts (except for the transmural ESPVR at 10 cmH_2_O PEEP) were also small but significant.

**Table 3:** Systolic impedance catheter data and ventriculo-aortic coupling

		*n*	Positive end-expiratory pressure			Friedman
			0 cmH_2_O	5 cmH_2_O	10 cmH_2_O	p value
Systolic function and inotropy						
Ejection fraction	%	25	69 ± 9	67 ± 9	69 ± 7	0.163*
End-systolic pressures	mmHg	25	123 ± 17	128 ± 20	126 ± 19	0.384*
D*p*/d*t*_max_	mmHg/sec	25	1252 ± 180	1230 ± 150	1206 ± 154	0.121*
Coupling						
Aortic elastance	mmHg/ml	25	1.16 ± 0.33	1.16 ± 0.37	1.14 ± 0.30	0.831*
Ventriculo-arterial coupling		25	1.95 (1.12 to 2.78)	1.88 (0.96–4.37)	1.99 (1.25 to 3.29)	0.326
Stroke work						
Stroke work	mL*mmHg		10,818 ± 2773	11,190 ± 2085	10,932 ± 2202	0.589*
Preload recruitable stroke work	mmHg		69 + 11	67 ± 10	64 ± 10	0.420*

The pressure reference is to atmosphere

*Normal distribution, one-way repeated measurements analysis of variance

**Table 4:** Linear mixed-effect model with interactions for ESPVR

	Coefficients	Std. err	*p* value	95% confidence interval	
ESPVR (atmospheric reference)					
Intercept for PEEP 0 cmH_2_O at mean volume (50.4 mL)	123.79	5.58	< 0.001	112.85 to 134.72	
* Changes in intercepts*					
PEEP 5 cmH_2_O	− 3.94	0.82	< 0.001	− 5.55 to − 2.32	
PEEP 10 cmH_2_O	2.24	0.89	0.012	0.49 to 3.97	
* Slope for PEEP 0 cmH*_*2*_*O*	1.05	0.11	< 0.001	0.85 to 1.26	
* Changes in slopes*					
PEEP 5 cmH_2_O	0.07	0.04	0.07	− 0.01 to 0.15	
PEEP 10 cmH_2_O	0.20	0.05	< 0.001	0.10 to 0.29	
ESPVR (transmural pressure)					
Intercept for PEEP 0 cmH_2_O at mean volume (50.4 mL)	117.08	5.42	< 0.001	106.46 to 127.70	
* Changes in intercepts*					
PEEP 5 cmH_2_O	− 6.46	0.91	< 0.001	− 8.25 to − 4.68	
PEEP 10 cmH_2_O	− 0.66	0.98	0.50	− 2.58 to 1.27	
* Slope for PEEP 0 cmH*_*2*_*O*	0.98	0.11	< 0.001	0.77 to 1.19	
* Changes in slopes*					
PEEP 5 cmH_2_O	0.06	0.04	0.18	− 0.03 to 0.15	
PEEP 10 cmH_2_O	0.19	0.05	0.001	0.08 to 0.30	

All interactions except 5 cmH_2_O PEEP are significant

*ESPVR* end-systolic pressure volume relationship, *PEEP* positive end-expiratory pressure

#### Relaxation

**Fig. 2:**
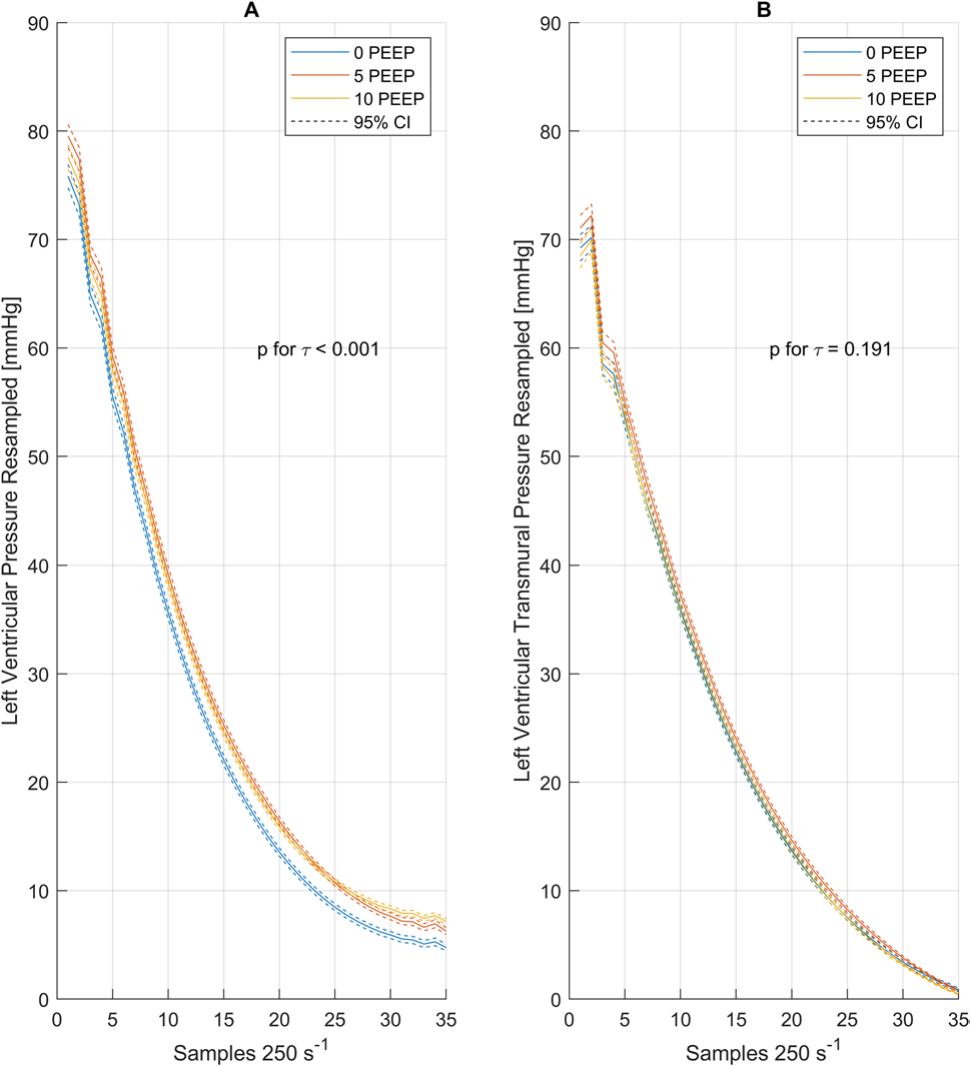
Mean left (**a**) and left transmural (**b**) ventricular pressures following an exponential decay model during isovolumetric relaxation, with 95% confidence intervals as dashed lines, resampled for 35 samples (140 ms). All data from all patients are included. PEEP, positive end-expiratory pressure

The application of PEEP significantly increased *τ* values for intracavitary ventricular pressures, indicating slower early relaxation of the left ventricle. This effect could be observed independently of the decay model used, and was sustained for the transmural cardiac pressures (Fig. 2, Table 5). When* τ* was corrected for the diastole length, the effect disappeared for the transmural pressure decay.

**Table 5:** Diastolic impedance catheter and modeled pressure decay

		*n*	Positive end-expiratory pressure			Friedman ANOVA
			0 cmH_2_O	5 cmH_2_O	10 cmH_2_O	*p* value
tau analysis						
* τ*_Glantz_	ms	25	43.6 (35.2–57.4)^*,†^	44.3 (33.7–58.7)	44 (34.2–58.5)	** < 0.001**
* τ*_Glantz,_ transmural	ms	25	46.8 (39.4–87.7)*	49.6 (41.1–75.3)	48.7 (37.5–70.8)	**0.025**
* τ*_logistic_	ms	25	28.7 (23.9–36.9)^*,†^	29.5 (22.7–37.6)	29.6 (23.5–37.1)	**0.001**
* τ*_logistic,_ transmural	ms	25	48.5 (27.2 to − 90.7)^*,†^	48.8 (30.2–135.2)	48 (25.8–100)	**0.01**
Normalized to length of diastole						
*τ*_Glantz_	ms	25	46 (38.4–54.4)	47.5 (37.5–55.2)‡	46.3 (34.4–55.3)	** < 0.001**
*τ*_Glantz,_ transmural	ms	25	61.5 (40.1–147)	62.3 (43.4–138)	66.7 (38–105.8)	0.191
*τ*_logistic_	ms	25	32.2 (26.3–49.3)	32.6 (25.7–64)^‡^	32 (23.6 to − 52.5)	**0.007**
*τ*_logistic,_ transmural	ms	25	48.5 (27.2–90.7)	48.8 (30.2–135.2)	48 (25.8–100)	0.225
INCA raw analysis						
*τ*_linear_	ms	25	31.6 (26.3–40)^*,†^	34.2 (28.7–43.1)	35 (30.3–44)	** < 0.001**
d*p*/d*t* min	mmHg/sec	25	− 1321 (− 1648 to − 933)	− 1385 (− 1787 to − 934)	− 1394 (− 1757 to − 843)	0.432
Diastolic filling time	ms	25	464 (302 to − 730)	511 (266–730)	528 (311–810)	**0.007**
Peak filling rate	mL/s	25	663 (352–1040)	631 (442–1128)	631 (437–1094)	0.527
Mid diastolic pressure at PFR	mmHg	25	6.6 (0.2–11.7)^*,†^	7.7 (− 0.5 to − 20.8)	9.8 (2.4–15.7)	**0.003**
EDP	mmHg	25	15.3 (6.1–27.5)	15.5 (4.4–26.8)	15.9 (8.4–26.7)	0.368
EDV	ml	25	166 (107–195)	177 (107–210)	159 (125–211)	0.102
ESP	mmHg	25	127 (90–155)	125 (93–173)	129 (87–157)	0.852
ESV	ml	25	57 (26 to − 93)	66 (25–105)	55 (36–109)	0.228

*p* < 0.05, post-hoc Wilcoxon test: *0 vs. 5 cmH_2_O, ^†^0 vs. 10 cmH_2_O, ^‡^5 vs. 10 cmH_2_O PEEP

*ANOVA* analysis of variance, *PFR* peak flow rate, *EDP* end-diastolic pressure, *EDV* end-diastolic volume, *ESP* end-systolic pressure, *ESV* end-systolic volume

*p*-values ≤0.05 were considered statistically significant and are indicated bold

#### Passive filling properties

**Fig. 3:**
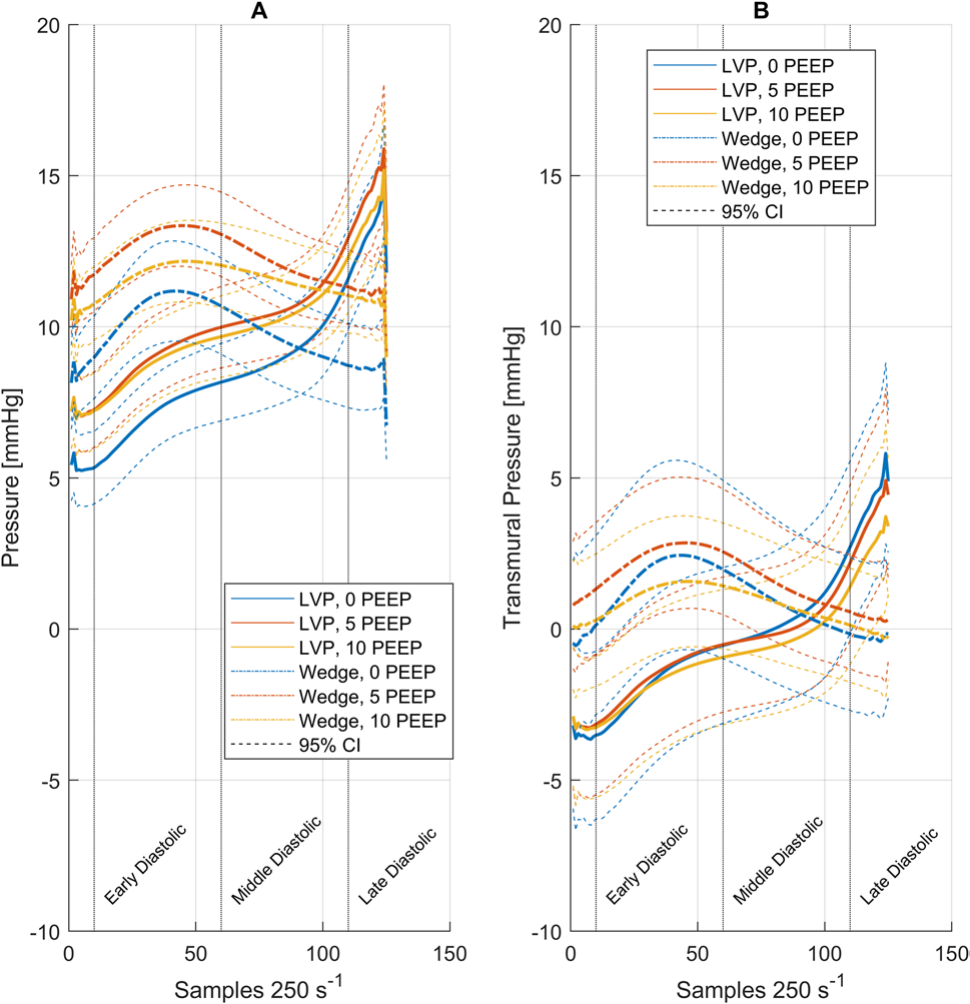
**a** Left ventricular filling curves represented by diastolic ventricular pressure and PAOP, with 95% confidence intervals as dashed lines. The data were resampled for a filling period of 125 samples (500 ms). The dotted lines for early, mid- and late diastolic data points indicate the data presented in Table 4. **b** Transmural left ventricular filling curves Means with 95% confidence intervals as dashed lines are presented. The data were resampled to a filling period of 125 samples (500 ms). The dotted lines for early, mid- and late diastolic data points. *PAOP* pulmonary-artery occlusal pressure, *LVP* left ventricular pressure, *PEEP* positive end-expiratory pressure

Early and mid-diastolic ventricular pressures and simultaneous pulmonary-artery occlusion pressures were significantly higher with PEEP (5 and 10 cmH_2_O) than without PEEP, with no difference between PEEP levels (Table 6, Fig. 3a). This effect disappeared when transmural pressures were calculated (Table 6, Fig. 2b). The instantaneous transmural trans-mitral pressure gradient remained unchanged with PEEP (Fig. 2).

**Table 6:** Diastolic left heart pressure properties

		*n*	Positive end-expiratory pressure			Friedman ANOVA		
			0 cmH_2_O		5 cmH_2_O	10cmH_2_O		*p* value
*Left ventricular pressure*								
Early diastolic	mmHg	24	5.2 (0.13–9.5)^*,†^		7.6 (1.2 to 12.8)	6.7 (− 0.35 to 12.5)		** < 0.001**
Early diastolic, transmural	mmHg	24	− 1.3 (− 22–4.3)		− 2.1 (− 21 to 5.6)	− 2.7 (− 9.9 to 2.7)		0.687
Mid diastolic	mmHg	25	8.9 (2.6–13.6)^*,†^		10.4 (3.0 to 16.6)	9.8 (0.9 to 14.6)		**0.001**
Mid diastolic, transmural	mmHg	24	1.6 (− 19.8–7.7)		0.35 (− 18.2 to 7.5)	− 0.93 (− 7.6 to 4.9)		0.417
Late diastolic	mmHg	25	11.6 (4.1–24)		12.8 (5.2 − 23.6)	12.1 (3.6 to 21.8)		0.167
Late diastolic, transmural	mmHg	24	2.7 (− 14.7–18)		2.2 (− 17.4–13.9)	1.4 (− 6.3–10.5)		0.197
*Pulmonary artery occlusion pressure*								
Early diastolic	mmHg	25	8.7 (− 0.2 to 13.8)^*,†^		11.9 (6.2–18.2)	10.4 (− 0.2 to 19.5)		**0.005**
Early diastolic, transmural	mmHg	25	2.1 (− 18.3 10.6)		1.5 (− 16 to 10)	0.9 (− 18.8 to 12.5)		0.846
Mid diastolic	mmHg	25	10.6 (− 0.2 to 18.8)^†^		12.5 (8.9–21.1)	12.6 (− 0.2 to 18.4)		**0.037**
Mid diastolic, transmural	mmHg	25	3.6 (− 14.8 to 12.9)		3.6 (− 14.4 to 8.9)	2.2 (− 18.8 to 12.9)		0.214
Late diastolic	mmHg	25	8.4 (− 0.23 to 16.6)^*,†^		10.4 (7.7 to 20.7)	11.2 (− 0.2 to 17.5)		**0.002**
Late diastolic, transmural	mmHg	24	0.4 (− 13.6 to 7.8)		1.7 (− 15.9 to 6.2)	0.7 (− 19.5 to 11.9)		0.747
*Transmitral pressure gradient*								
Early diastolic	mmHg	24	3.7 (− 7.6 to 13.7)		4.5 (− 1.7 to 11.3)	3.8 (− 8.9 to 13.8)		0.687
Mid diastolic	mmHg	24	2.9 (− 10.9 to 7.6)		2.9 (− 2.3 to 9.2)	2.9 (− 11.4 to 13.2)		0.607
Late diastolic	mmHg	24	− 3.0 (− 13.1 to 3.9) ^†^		− 1.2 (− 9.5 to 4.9)	− 0.9 (− 13.2 to 9.6)		**0.048**

*p* < 0.05, post-hoc Wilcoxon test: *0 vs. 5 cmH_2_O, ^†^0 vs. 10 cmH_2_O, ^‡^5 vs. 10 cmH_2_O PEEP

*ANOVA* analysis of variance

*p*-values ≤ 0.05 were considered statistically significant and are indicated bold

#### Positions of the EDPVR and chamber stiffness

**Fig. 4:**
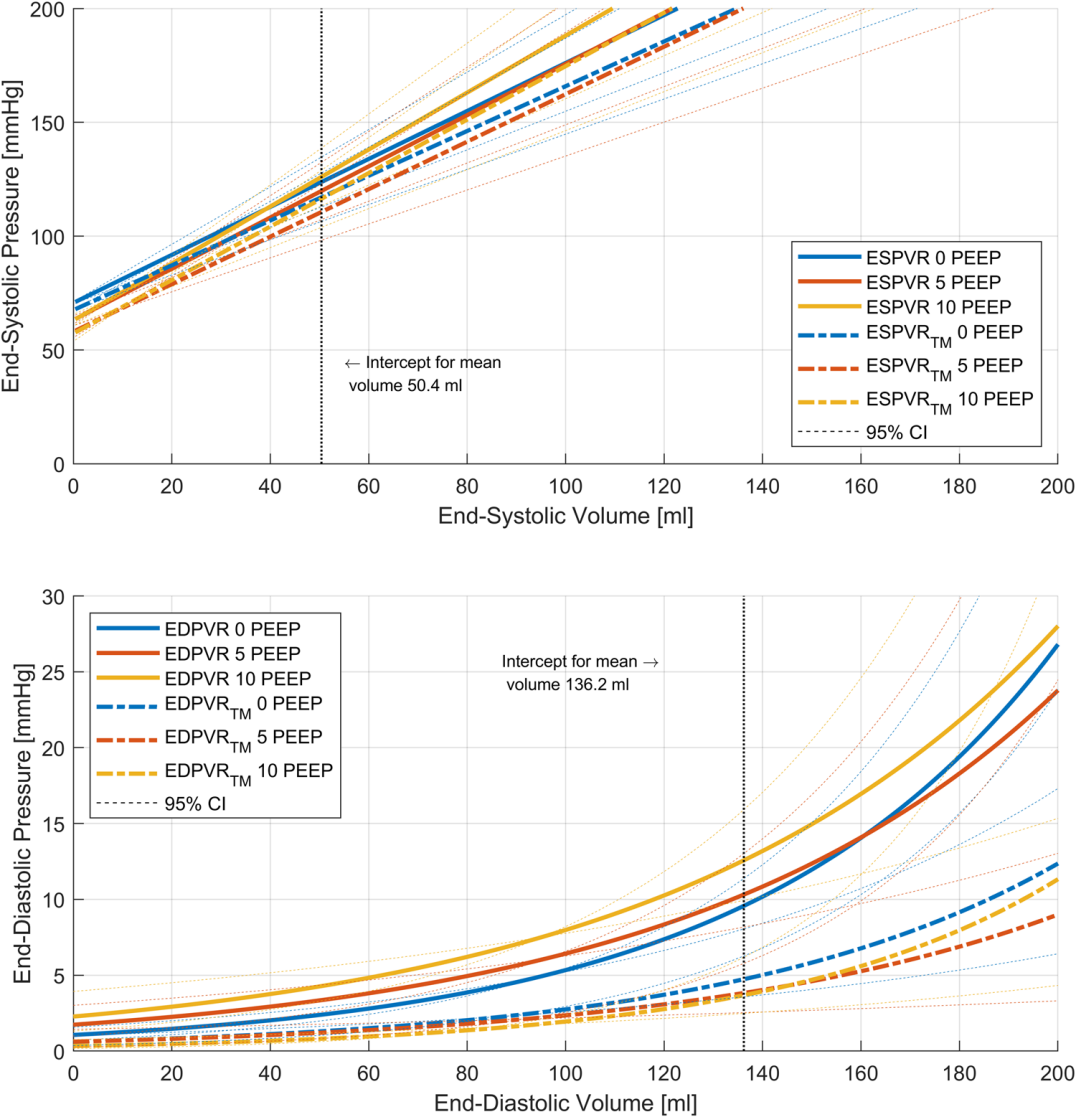
**a** End-systolic pressure–volume relationships (ESPVRs) for 0, 5, and 10 cmH_2_O PEEP, according to the mixed linear model equations in Table 4. The equation values are reported at the mean centered volume (50.4 ml), indicated by the dotted line. **b** End-diastolic pressure–volume relationships (EDPVRs), according to the mixed-effect generalized linear model equations in Table 7. The equation values are reported at the mean centered volume (136.2 ml), indicated by the dotted line. *PEEP* positive end-expiratory pressure

The generalized linear mixed-effects model revealed small, but significant, changes in the natural logarithms of the intercept changes in the absolute and transmural EDPVRs. The resulting intercept pressures from the inverse function (for intracavitary pressures: no PEEP, 9.57 mmHg; 5 cmH_2_O PEEP, 10.31 mmHg; 10 cmH_2_O PEEP, 12.57 mmHg; for transmural pressures, no PEEP 4.73 mmHg, 5 cmH_2_O PEEP, 3.82 mmHg; 10 cmH_2_O PEEP, 3.68 mmHg) indicate a downward shift of the EDPVR for the transmural pressure, with a resolution of between-PEEP differences. The change in slope, representing the chamber stiffness, lost significance when corrected for transmural pressure (Table 7). The shift in position and change in stiffness with increasing PEEP disappeared when transmural pressures were considered (Fig. 4).

**Table 7:** Generalized linear mixed-effects model for EDPVR

	Coefficients	Std. err	*p* value	95% Confidence interval
EDPVR (atmospheric reference)				
ln Intercept for PEEP 0 cmH_2_O at the mean volume (136.2 mL)	2.26	0.09	< 0.001	2.09–2.43
Changes in ln of intercepts				
PEEP 5	0.08	0.03	0.015	0.01–0.14
PEEP 10	0.27	0.03	< 0.001	0.21–0.34
ln Slope coefficient PEEP 0 cmH_2_O	0.02	0.00	< 0.001	0.01–0.02
Changes in ln of slope coefficients				
PEEP 5 cmH_2_O	− 0.003	0.0008	< 0.001	− 0.005 to − 0.001
PEEP 10 cmH_2_O	− 0.004	0.0008	< 0.001	− 0.005–0.002
EDPVR (transmural pressure)				
ln Intercept for PEEP 0 at the mean volume (136.2 mL)	1.55	0.14	< 0.001	1.27–1.84
Changes in ln of intercepts				
PEEP 5	− 0.21	0.07	0.001	− 0.34 to − 0.08
PEEP 10	− 0.25	0.06	< 0.001	− 0.37 to − 0.14
ln Slope coefficient for PEEP 0	0.02	0.003	< 0.001	0.009–0.02
Changes in ln of slope coefficients				
PEEP 5	0.002	0.002	0.337	− 0.005 to 0.002
PEEP 10	0.003	0.002	0.094	− 0.0004 to 0.006

All values are given as natural logarithms

*EDPVR* end-diastolic pressure volume relationship, *PEEP* positive end-expiratory pressure

### Echocardiographic diastole evaluation

#### Trans-mitral flow velocities

**Fig. 5:**
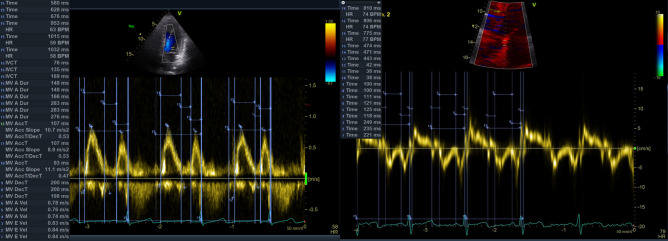
Exemplary echocardiography tracings at a PEEP level of 10 cm H_2_O. The frames that correspond to the PV-Loop family in Fig. 1 are shown. The left panel shows a transmitral pulsed wave Doppler trace, the right panel a tissue Doppler tracing

The peak early and late trans-mitral flow velocities (*E* and *A*) decreased significantly with increasing PEEP, with no change in the *E*/*A* ratio. The duration of the E wave increased in absolute terms, but remained constant relative to the duration of diastole and the full cardiac cycle. The duration of the A wave and the deceleration time remained constant (Table 5, Fig. 5).

#### Tissue Doppler properties of the mitral annulus

The peak early septal tissue velocity (*E*ʹ) decreased with PEEP. The lateral *E*ʹ and late tissue velocities (*A*ʹ) were less affected. The isovolumetric relaxation time increased with PEEP (Table 8).

**Table 8:** Echocardiographic diastology parameters

		*n*	Positive end-expiratory pressure			Friedman
			0cmH_2_O	5cmH_2_O	10cmH_2_O	*p* value
*PW Doppler analysis mitral flow*						
*E*	m/s	25	0.77 (0.53–1.04)^†^	0.76 (0.49–1.05)	0.66 (0.5–0.94)	**0.016**
DecT	ms	25	189 (111–300)	183 (131–331)	187 (114–306)	0.756
*A*	m/s	24	0.73 (0.53–1.02)^†^	0.71 (0.5–0.99)^‡^	0.63 (0.44–1)	**0.014**
*E*/*A*		23	1.12 (0.62–1.45)	1.05 (0.66–1.56)	1.09 (0.66–1.38)	0.438
*R*–*R*-interval	ms	25	956 (717–1194)^*,†^	993 (765–1259)	960 (758–1205)	**0.026**
Diastole	ms	25	555 (357–790)^*,†^	606 (384–853)	600 (376–806)	**0.001**
Diastole/* R*–*R*-interval	%	25	58 (49–66)^*,†^	62 (50–68)	61 (48–72)	**0.006**
Edur	ms	22	251 (168–362)^†^	252 (188–398)	261 (176–379)	**0.036**
Edur/*R*–*R*-interval	%	22	25.2 (18.6–35.1)	24.7 (20–37.6)	26.6 (20.1–35.7)	0.834
Edur/diastole	%	22	44.5 (30.1–55)	41.4 (32–55.3)	44.1 (31.2 (54.7)	0.554
Adur	ms	22	131 (94–161)	137 (94–162)	135 (101–166)	0.386
Adur/*R*–*R*-interval	%	22	14.3 (7.9–16.4)	13.6 (8.6–17.4)	13.7 (9.5–15.8)	0.58
Adur/diastole	%	22	24.4 (11.9–30)	22 (13.6–29.7)	22.6 (14.3–28.4)	0.554
*Tissue Doppler mitral annulus*						
*E*ʹ septal	m/s	24	0.082 (0.06–0.13)^*,†^	0.08 (0.06–0.12)	0.076 (0.05–0.1)	**0.002**
*E*ʹ lateral	m/s	25	0.11 (0.08–0.15)	0.11 (0.076 to 0.13)	0.11 (0.06–0.16)	0.647
*E*/*E*ʹ mean		23	7.2 (5.5–11.8)	8.0 (5.3–10.8)	7.4 (4.4–13.2)	0.17
*R*–*R*-interval	ms	24	963 (732–1165)	991 (743–1203)	993 (780–1265)	0.197
*A*ʹ septal	m/s	24	0.085 (0.046–0.14)	0.086 (0.06–0.12)	0.080 (0.04–0.13)	0.3
*A*ʹ lateral	m/s	25	0.096 (0.07–0.14)^†^	0.0.093 (0.06–0.13)	0.083 (0.05–0.16)	**0.01**
IVRT	ms	24	66.7 (47–92.3)^*,†^	79 (34.6–102.6)	82 (48–115.3)	**0.009**
IVRT/*R*–*R*-interval	%	24	7 (4.7–9.5)^†^	7.8 (3.8–11.2)	8.2 (5.1–13)	**0.01**

*p* < 0.05, post-hoc Wilcoxon test: *0 vs. 5 cmH_2_O, ^†^0 vs. 10 cmH_2_O, ^‡^5 vs. 10 cmH_2_O PEEP

*E* early ventricular filling wave, *DecT* deceleration time, *A* atrial ventricular filling wave, *Edur* E wave duration, *Adur* A wave duration, *IVRT* isovolumetric relaxation time, *PW* pulse wave Doppler mode

*p*-values ≤0.05 were considered statistically significant and are indicated bold

## Discussion

The main finding of this study is that the increase in intrathoracic pressure caused by mechanical ventilation had no intrinsic influence on cardiac function, assessed invasively by the gold standard (impedance catheter). Inotropy, represented by the ESPVR, increased slightly with PEEP. The invasive assessment of diastolic function revealed a small influence of PEEP on active relaxation, with an apparent increase in *τ*, upward shift of the EDPVR, and decreasing chamber stiffness, indicating altered passive ventricular filling and elastic properties, but only when the pressures were referenced to atmosphere [11]. These changes in invasive measures disappeared with the measurement of transmural chamber pressures, which reflect the working condition of the left ventricle. These results stand in contrast to echocardiographic parameters, as modest PEEP influences the transmitral flow pattern and annular tissue Doppler properties.

These findings challenge current paradigms and must be placed in clinical and physiological contexts. The inaccuracy of transmitral indices for the assessment of diastolic dysfunction has long been discussed [9] 30. Comparative Doppler-conductance catheter studies with non-ventilated patients have shown that tissue Doppler indices may better reflect diastolic dysfunction than do transmitral flow velocities, and that increasing E/E’ ratios are the best indicators of diastolic dysfunction [30, 31]. Our data, support this notion. The load dependencies of transmitral *E* and *A* waves are recognized [17], albeit not in comparison with invasive assessment under positive-pressure ventilation. Juhl-Ohlsen and colleagues . [15, 16] demonstrated the influence of PEEP on *E* and *A* waves in anesthetized cardiac patients. PEEP increases the pleural pressure [20], explaining its well-established preload-reducing effect [11, 21, 25, 32]. Our echocardiographic findings are in line with these previous observations. The *E*/*E*ʹ ratio, strongly predictive of ventilator weaning failure [3], was not affected by PEEP in our population.

We observed apparent slowing of active relaxation, with increases in *τ* with the application of PEEP. Despite its significance in the intracavitary pressure assessment, the magnitude of this change appears to be clinically marginal. For its proper interpretation, two physiological phenomena need to be taken into account. The first is the heart rate, and thus the duration of diastole. Several studies have shown that *τ* declines with increasing heart rate and vice versa [33–35]. As the heart rate changes with PEEP, we normalized the diastole duration in a second model of *τ* to exclude influences of changing diastole length in this study. The second issue is the exposure of the heart to pleural, rather than atmospheric, pressure [11]. In routine cardiac catheterization and hemodynamic monitoring, intracavitary and intravascular pressures are measured with a zero reference to the atmosphere. The pericardial pressure, however, approximates the pleural pressure [36]. Cardiac working conditions are better represented by transmural rather than intracavitary pressures. The subtraction of the esophageal pressure from intracavitary pressures may serve as a sufficient approximation [11, 20, 36–38]. When this intrathoracic (i.e., pleural) pressure reference is taken into account, the apparent effect of PEEP on *τ* disappears. Thus, findings may reflect the use of an inappropriate pressure reference, rather than the occurrence of a true physiological phenomenon. The consistency of the findings obtained with various modeling approaches for *τ* reflects reliability, particularly as the logistic modeling of pressure decay is relatively resistant to respiration influences [28]. In conductance volumetry, unchanged ventricular volumes may exclude ventricular interdependence as a cause of impaired relaxation [39].

PEEP applies a mechanical constraint to the heart by increasing the pleural pressure and elevating the functional residual capacity of the lung, with compression of the cardiac fossa [40]. Increased pericardial pressure shifts the pressure–volume curve upward [41], and a similar shift in the EDPVR slope was observed in this study. This upward shift, and the apparent decrease in chamber stiffness, disappeared when transmural pressures were considered. These findings may thus be considered artifacts of the use of the atmospheric pressure reference. The clinically modest PEEP levels used in this study had no apparent effect on cardiac chamber volumes; shifts may differ with the application of greater pressure, particularly with invasive mechanical ventilation [42]. We may only speculate on the effects of higher PEEP, as the preload reducing effect together with mechanical constraint may dominate afterload reduction and resemble restrictive filling with signs of obstructive shock [11]. Such data would need to be gained from invasive ventilation because higher pressure levels on NIV are usually badly tolerated.

The slope of the ESPVR at 10 cm H_2_O PEEP increased, with a positive (intracavitary) or no (transmural) relevant increase in the pressure intercept, in this study. These findings are consistent with an increased inotropic state of the left ventricle [29]. At 5 cmH_2_O PEEP, the increase in slope was less prominent and of borderline significance. We have no mechanistically convincing explanation for this unexpected result. Data from animal studies suggest that PEEP negatively affects the coronary blood flow [43], which would have the opposite effect as we observed. The Anrep effect, or slow force response to increased stretch, could explain the increased inotropy [44, 45], but the afterload seems to be constant in our context with stable ventriculo-arterial coupling across PEEP levels. Verification of the direct positive inotropic effects of PEEP would provide an additional mechanistic explanation of the beneficial effects of positive pressure ventilation for acute cardiac failure, beyond the classic concept of preload and afterload reduction.

## Limitations

Several limitations of this study must be considered. First, the study participants were cardiac patients with no apparent diastolic dysfunction at the time of enrollment, as verified by echocardiographic grading and low *τ* values on spontaneous breathing. Whether similar results would be obtained in patients with such dysfunction or those under invasive mechanical ventilation in an intensive care unit setting warrants further investigation. The advantage of conducting the study with patients without diastolic dysfunction is that we could demonstrate the appearance of such dysfunction with echocardiography. We can only speculate about effects of PEEP in patients with preexisting diastolic function. These effects may be less unidirectional, since the mechanical constraint caused by PEEP may be counterbalanced by its afterload reducing effect in the clinical context of heart failure with preserved ejection fraction or obstructive sleep apnea. Clinical studies indicate long term amelioration of diastolic dysfunction with positive pressure ventilation [46, 47].

Second, our echocardiographic finding of increasing diastolic dysfunction severity with increasing airway pressure may be clinically elusive. Its importance lies not in its magnitude, but in its difference from the conductance-catheter gold standard. Third, voluntary control of respiration in an awake person under an invasive hemodynamic study is demanding. We took meticulous care to instruct the participants in performing expiratory holds and monitored their respiratory drives with esophageal pressure swings. An attending intensivist supervised all maneuvers, and the esophageal pressure swings were screened for inadvertent Valsalva maneuvers. Still, unintended respiratory variation cannot be excluded. Thus, the use of *τ* logistic, which is least influenced by respiration [28], was of particular value. Fourth, an esophageal balloon is the only feasible pleural pressure surrogate in an awake patient and its use is recommended [38], although this technique may not directly reflect pericardial pressure. We have experience in esophageal balloon use [20, 25], and calibrated the balloon using an accepted technique in this study [37, 38]. As each patient served as his/her own control in the randomized crossover setting, the esophageal pressure was a valid relative reference, although absolute values might have been slightly inaccurate. Fifth, calibration of the PV loops required a two-step approach. The stroke volume was calibrated via thermodilution at every PEEP level. We followed a standardized approach for thermodilution by averaging the three closest cardiac output measurements from five saline injections [48]. The PV loops were positioned on the volume axis by calculating the end-diastolic volume from a baseline ejection fraction measurement (obtained by echocardiography or with hypertonic saline injection). The variability of the PV loop position through the ejection fraction may have contributed to shifts in intercept and slope, but was partially accounted for with the use of mixed-effects models for the ESPVR and EDPVR. Last, our sample size could not be based on a power calculation, because of the exploratory nature of this study and the lack of preexisting data.

## Conclusion

We conclude that dynamic echocardiographic filling parameters reflect changing loading conditions, rather than diastolic function, under the application of positive airway pressure. Furthermore, invasive assessments should be referenced to intrathoracic pressure conditions, rather than to atmospheric pressure. The role of echocardiography in the detection of diastolic dysfunction in mechanically ventilated patients needs to be reassessed.

## Supplementary Information

Below is the link to the electronic supplementary material.

Supplementary file1 (DOCX 8685 kb)

Supplementary file2 (DOCX 8659 kb)

## Data Availability

The datasets used and/or analyzed during this study are available from the corresponding author on reasonable request.

## Abbreviations

A: Peak late mitral inflow velocity
CPAP: Continuous positive airway pressure
DT: Deceleration time
E: Peak early mitral inflow velocity
E’: Peak early diastolic mitral annular velocity
Ea: Arterial elastance
Ees: End-systolic elastance
EDPVR: End-diastolic pressure–volume relationship
EDV: End-diastolic volume
EDP: End-diastolic pressure
EF: Ejection fraction
ESP: End-systolic pressure
ESPVR: End-systolic pressure–volume relationship
ESV: End-systolic volume
IVRT: Isovolumic relaxation time
LV: Left ventricle
LVEDP: Left ventricular end-diastolic pressure
PAOP: Pulmonary artery occlusion pressure
PEEP: Positive end-expiratory pressure
PFR: Peak filling rate
PW: Pulsed wave Doppler mode
SV: Stroke volume
TAPSE: Tricuspid annular plane systolic excursion
TDI: Tissue Doppler imaging
