# Supplementary material for: The effects of positive end-expiratory pressure on cardiac function: a comparative echocardiography-conductance catheter study
Source: Clin Res Cardiol. 2022 Apr 6;111(6):705–19. doi: 10.1007/s00392-022-02014-1 (PMC9151717; doi:10.1007/s00392-022-02014-1)
Supplement: Supplementary file 1 — Supplementary file1 (DOCX 8685 kb) [file 392_2022_2014_MOESM1_ESM.docx]

**The effects of positive end-expiratory pressure on cardiac function: a comparative echocardiography-conductance catheter study**

**Additional file 1**

**Expanded materials and methods**

**Inclusion and exclusion criteria**

Eligible participants had no known heart disease (normal ejection fraction, no valvular heart disease > grade 1, no previous myocardial infarction or open heart surgery, no pulmonary hypertension, no diastolic dysfunction > grade 1, sinus rhythm without bundle branch block), normal renal function, and no obstructive or restrictive pulmonary disease. Poor echocardiographic imaging quality or esophageal disorders were exclusion criteria, as was the need for aorto-coronary bypass surgery or multivessel percutaneous coronary intervention (PCI).

**Coronary angiography and PCI**

Coronary angiography, left ventricular (LV) angiography, and aortic and LV pressure assessment were performed following the standard procedure of the laboratory using a 5 French (F) right femoral arterial sheath and diagnostic catheters. Any required PCI was performed prior to the study-specific assessments and was limited to single-vessel treatment.

**Randomization**

A random generator and closed envelopes with pre-specified positive end-expiratory pressure (PEEP) sequence assignments were used to consecutively assign each patient to one of six PEEP sequence groups (five patients per group; 0, 5, and 10 cmH_2_O; 0, 10, and 5 cmH_2_O; 5, 0, and10 cmH_2_O; 5, 10, and 0 cmH_2_O; 10, 0, and 5 cmH_2_O; 10, 5, and 0 cmH_2_O).

**Noninvasive ventilation settings**

A facemask of appropriate size for noninvasive ventilation (ResMed full face mask, ResMed Schweiz AG, Liebefeld, Switzerland) was installed and connected to a ventilator (Servo-i; Maquet, Solna, Sweden). Its position was adapted to minimize air leakage, and time was allowed for the patient to become familiar with the mask and assisted breathing. Noninvasive ventilation was then initiated at an inspired oxygen fraction of 0.21. The trigger sensitivity was set to 5 l/min (flow trigger), the cycling off criterion was set at 40% of inspiratory flow, and the inspiratory rise time was set by default to 0.15, with adaptation to optimize patient comfort.

**Study-related assessment and course of maneuvers**

A nasogastric tube with an esophageal pressure balloon (Nutrivent esophageal pressure sensor; Sidam s.r.l., Mirandola, Italy) was placed[1]. Balloon volume calibration was performed as proposed by Mojoli [2]. A pulmonary-artery balloon catheter (Model 114F7P; Edwards Lifesciences, Irvine, California) was positioned in the right pulmonary artery under fluoroscopic guidance.

Through a 9F sheath, an Amplatzer Sizing Balloon II (34 mm, St. Jude Medical, St. Paul, Minnesota) was advanced to the conjunction of the inferior vena cava and the right atrium. Through the arterial sheath, a 7F 12-electrode dual-field conductance catheter (Sentron, Roden, The Netherlands) with 8- or 10-mm spacing between the sensing electrodes, depending on the LV size, was advanced across the aortic valve, placed in the left ventricle, and calibrated following connection to a signal processor (Sigma-5 DF; CD Leycom, Hengelo, The Netherlands), as described previously [3, 4]. A personal computer–based data acquisition system (INCA; CD Leycom, Hengelo, Netherlands) was used to record and analyze the electrocardiographic and LV pressure and volume signals and the right atrial, pulmonary artery, esophageal, and airway pressures. All pressures were measured using commercially available pressure transducers (x-trans; Codan, Lensahn, Germany) and fed into the data acquisition system that was used for the pressure-volume loops (INCA; CD Leycom) for local storage.

The VO_2_ was calculated based on the participant’s body surface area, sex, and age, according to LaFarge[5]. For each of the three ventilator settings (spontaneous breathing, low and high level positive pressure ventilation), the following procedure was performed:

1. Adjust ventilator settings, then wait 3 min or until the patient has adapted comfortably to the new setting

2. Take blood samples (2 mL each) from the pulmonary artery and left ventricle for blood gas analysis

3. Calibrate all pressure lines

4. Under end-expiratory breath holds:

- Simultaneously record the pulmonary-artery occlusion pressure and left ventricular pressure in parallel with transthoracic echocardiography (TTE) examination of mitral inflow, mitral tissue velocity, and event timing
- Simultaneously record lef ventricular pressure-volume loops and right atrial, pulmonary artery, esophageal and airway pressures
- Perform TTE as described below

5. Perform end-expiratory occlusion of the inferior vena cava and concomitant left ventricular pressure-volume loop, record right atrial and pulmonary artery pressures

6. Measure arterial blood pressure (arm cuff)

**Data collection and analysis**

*TTE*

TTE was performed using a Vivid S6 device with an M4S probe (GE Healthcare, Glattbrugg, Switzerland). Loops and frames were stored locally in raw data format on the hard disk of the echocardiography system. The following data were acquired at each setting: apical four- and two-chamber views, pulsed-wave Doppler data on mitral inflow, continuous-wave Doppler data from the left ventricular outflow tract, tissue Doppler images from the septal and lateral mitral annuli and lateral tricuspid annulus, and M-mode images of the lateral tricuspid annulus.

A cardiologist blinded to the ventilator settings evaluated the raw data offline using EchoPac software (Version 08; GE Medical Systems CH, Glattbrugg, Switzerland). The following parameters were measured for each ventilator setting:

a) from mitral inflow pulsed-wave Doppler and mitral annular tissue Doppler recordings, the early (E) and late (A) mitral inflow velocities and duration, deceleration time, and isovolumic relaxation and contraction time, and the systolic (S’), early (E’), and late (A’) diastolic mitral annular velocities and durations from the septal and lateral annuli; E/E’ was calculated using the mean septal and lateral mitral annular velocities;

b) from tricuspid annular tissue Doppler imaging, S’;

c) from M-mode images, the tricuspid annular plane systolic excursion;

d) from apical two- and four-chamber views, the biplane LV end-diastolic and end-systolic volumes (EDV and ESV, respectively) and ejection fraction (EF; calculated using the Simpson method).

*LV conductance catheter measurements*

Data files were stored on the local hard disk of a personal computer. The following parameters were assessed.

a) for each caval occlusion maneuver, the end-systolic and end-diastolic pressure–volume relationships and end-systolic and arterial elastance;

b) for each ventilator setting, the stroke volume (SV), cardiac output (CO), ESV, EDV, end-systolic pressure, end-diastolic pressure, EF,  volume and pressure changes over time (dV/dt_min&max_, dP/dt_min&max)_, tau (linear model), peak filling rate, dV/dP, stroke work, and preload recruitable stroke work.

As automated custom software for available pressure-volume loop systems allows for the assessment of only intracavitary pressures and has been criticized for inaccuracies, manual assessment is recommended [2]. We extracted the data from the INCA system into Matlab (Mathworks, Natick,Massachusetts) for the assessment of transmural pressures. The esophageal pressure was subtracted from the left ventricular pressure to obtain real-time transmural pressure values. Because of swings in the esophageal pressure measurements, we used median values to assess transmural end-diastolic pressure–volume relationships. The end-diastolic pressure and EDV points were defined using echocardiographic triggering with the QRS complex defining the beginning of each new systole. The end-systolic pressure and ESV points were defined using an algorithm in each loop to search for the maximum volume and beginning of pressure decay. The data points were then confirmed visually and filtered using an outlier removal algorithm.

The diastolic pressure decay was modeled with an exponential and logistic mathematical model [6]. The respective diastolic pressure segments were cut from the point of the dp/dp minimum (i.e., the minimum value of the first pressure time derivative, representing the steepest portion of the curve) to the minimum recorded pressure. Using that point in time for each cardiac sample, the passive filling curves were identified from minimal pressure up to the beginning of the next systole. Ventricular extrasystoles were excluded from the analysis.

**Individual patient data**

**Figure legend**

**Fig. 1** The top row shows the pressure decay of individual heartbeats with the corresponding τ values for the exponential (τ_Glantz_) and logistic (τ_log_) models. The middle row shows the transmitral flow gradient. The bottom row shows pressure-volume loops recorded during the caval occlusion maneuver; only those with at least three consecutive beats with decreasing volume and pressure uninterrupted by extrasystoles were included. Data in the left and right columns are intracavitary and transmural pressures, respectively, and their derivatives.

**Patient 1**
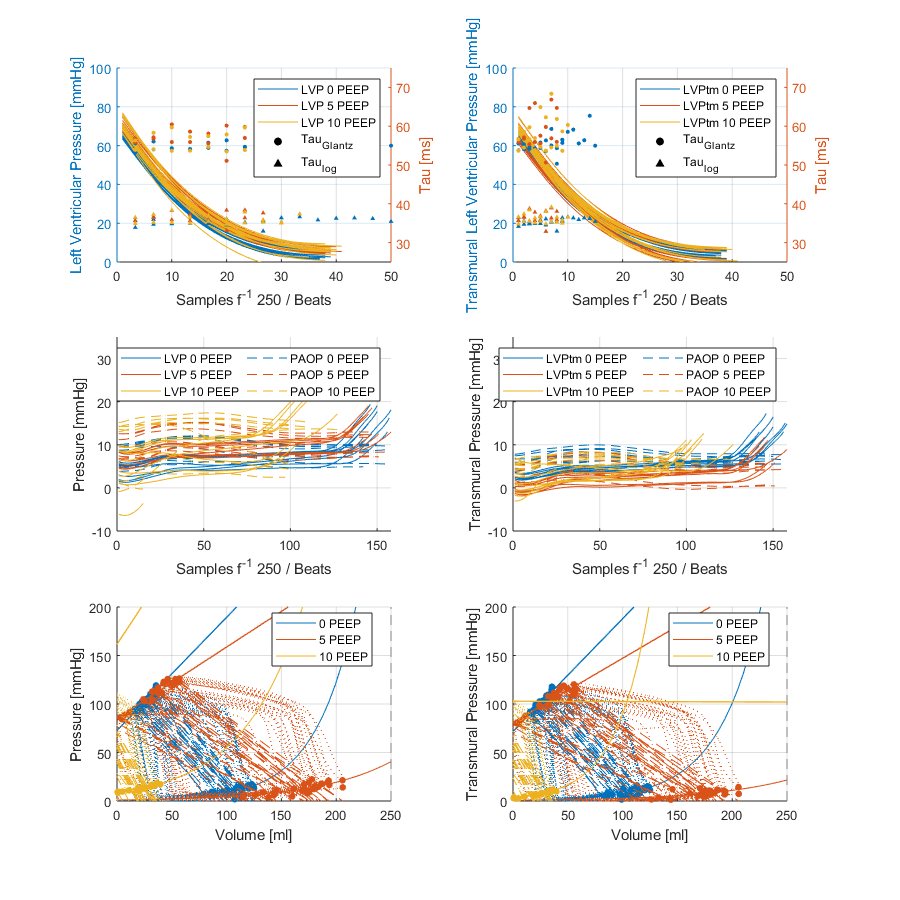


**Patient 2**


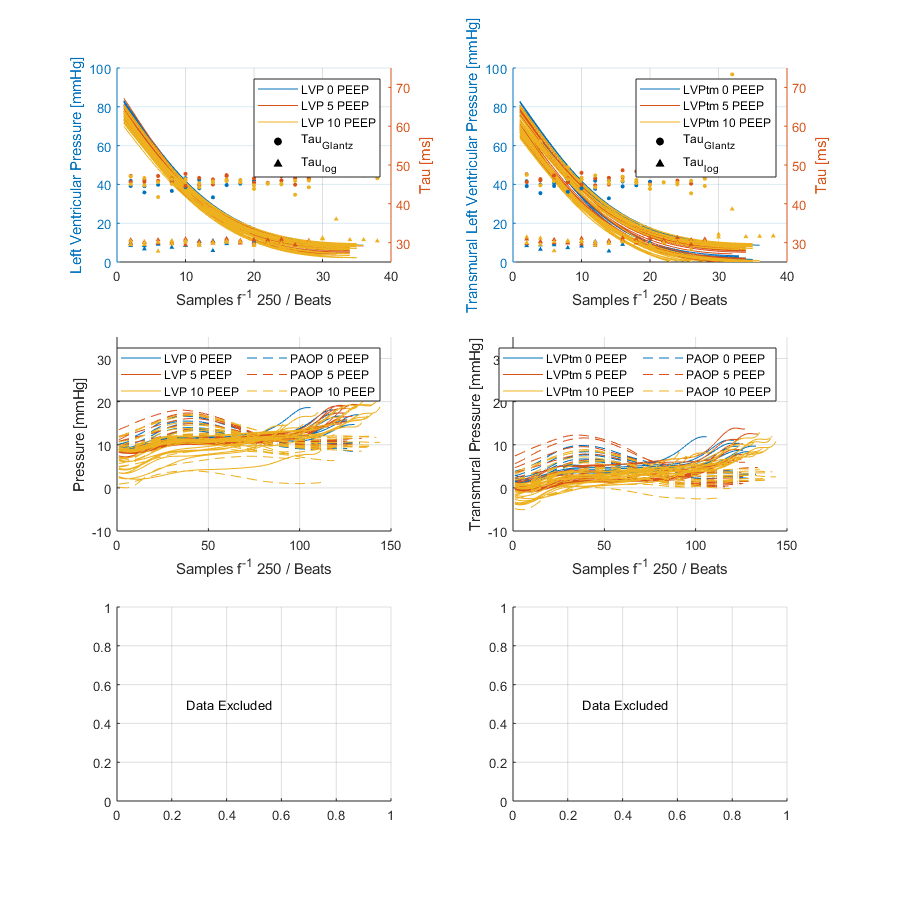


**Patient 3**


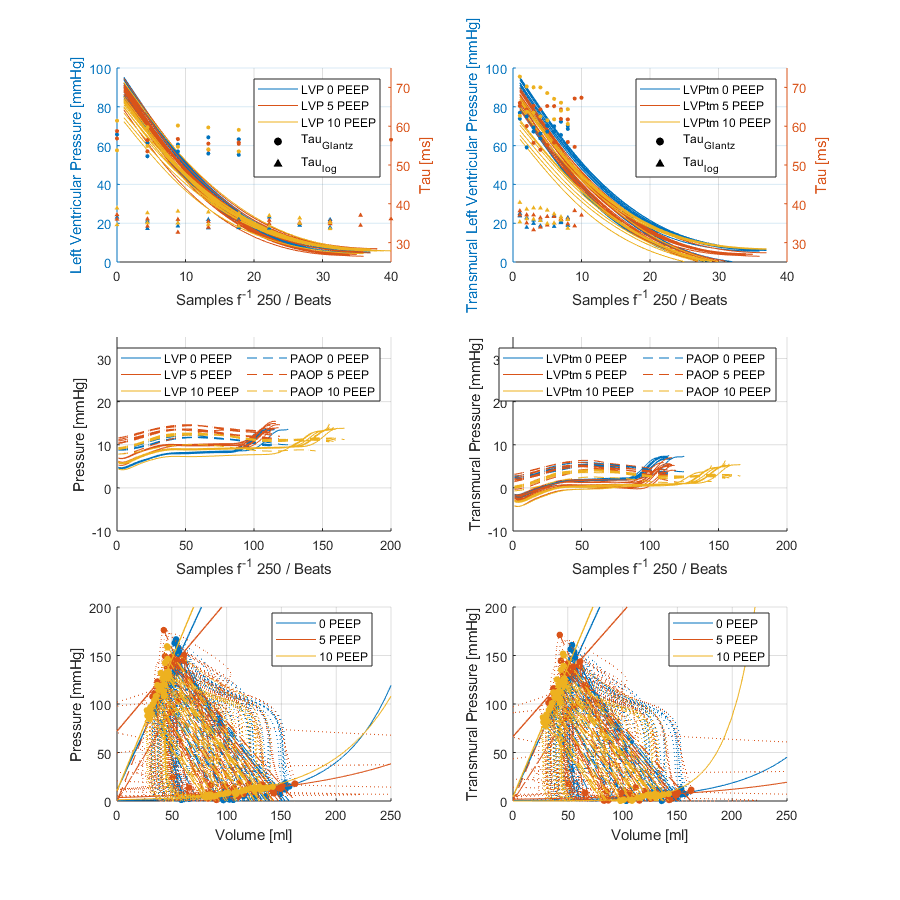


**Patient 4**


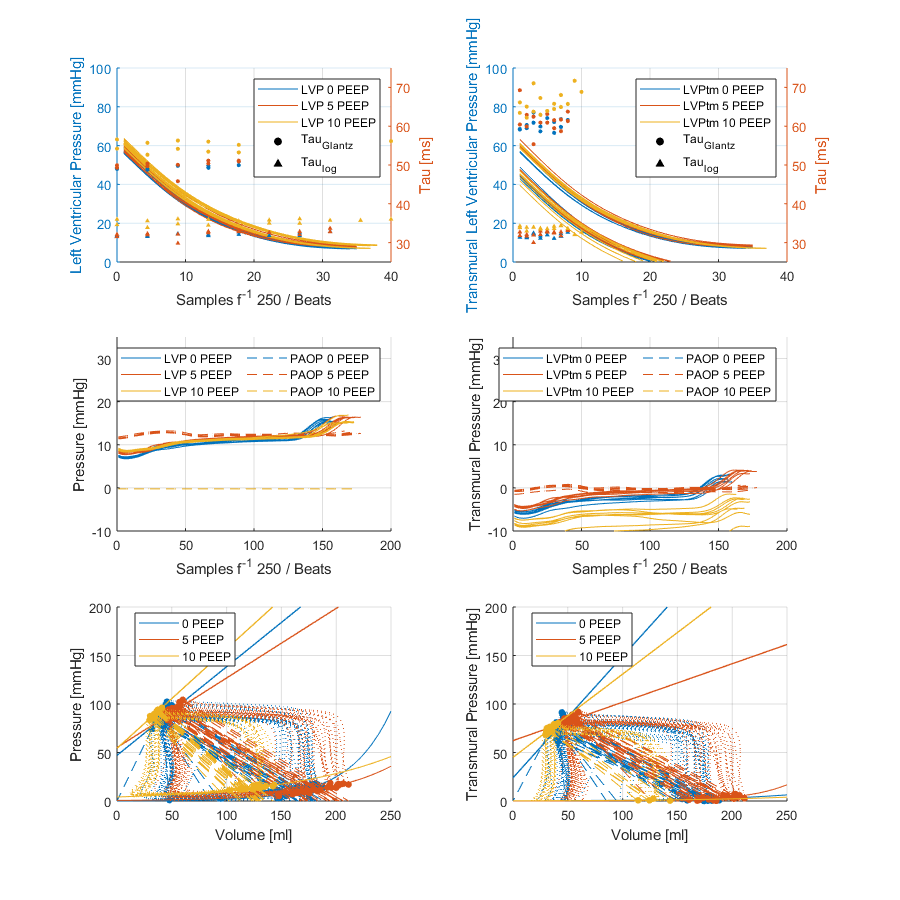


**Patient 5**


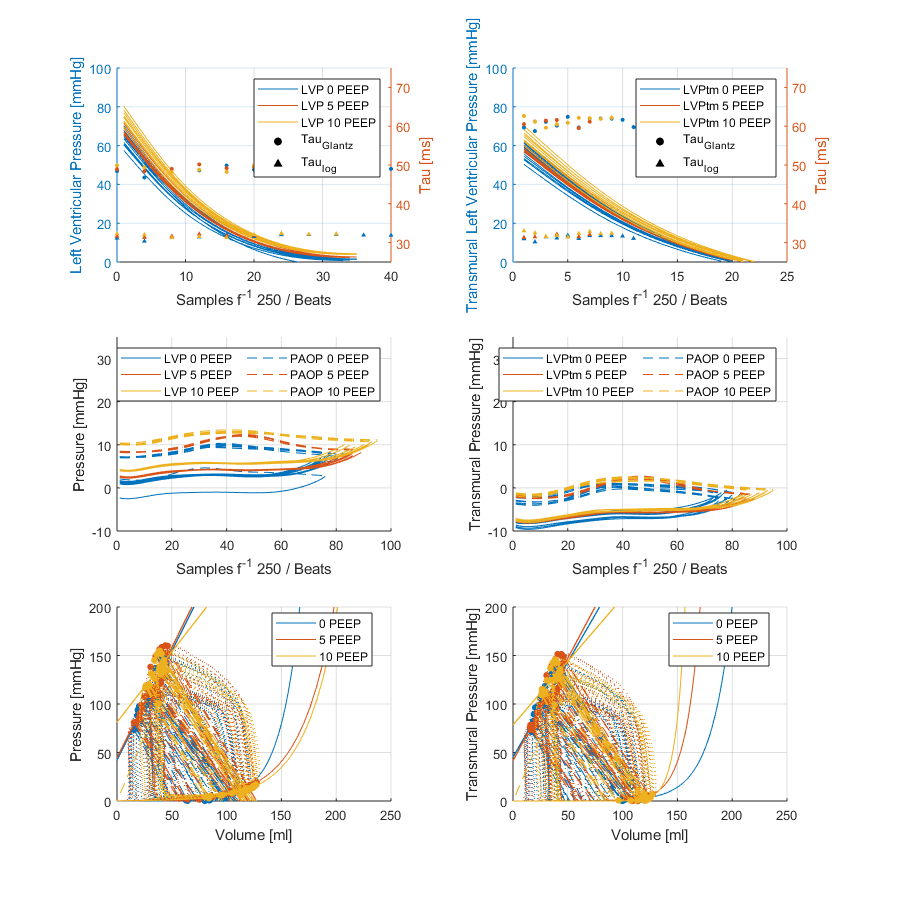


**Patient 6**


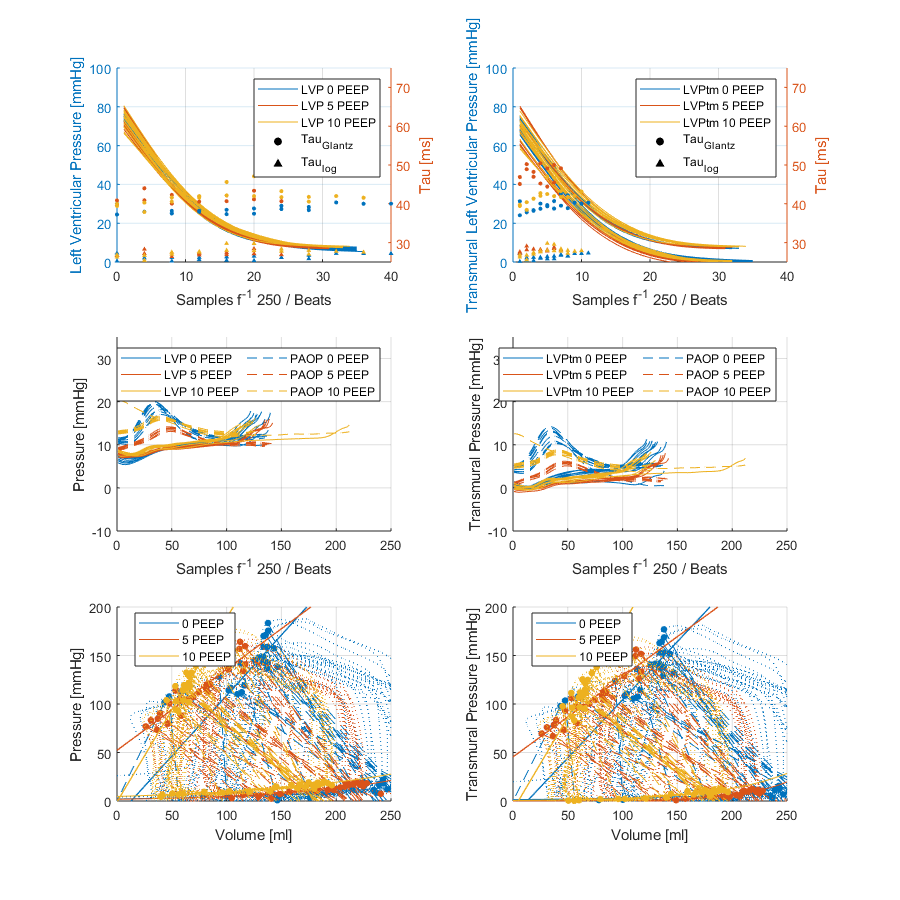


**Patient 7**


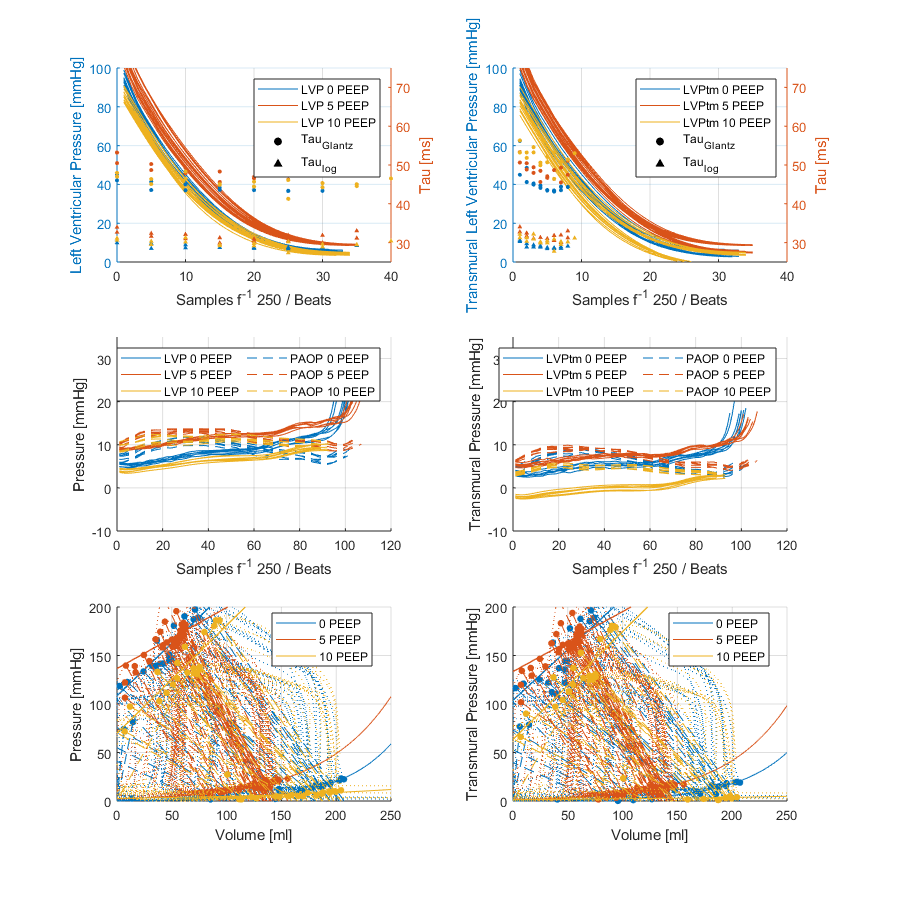


**Patient 8**


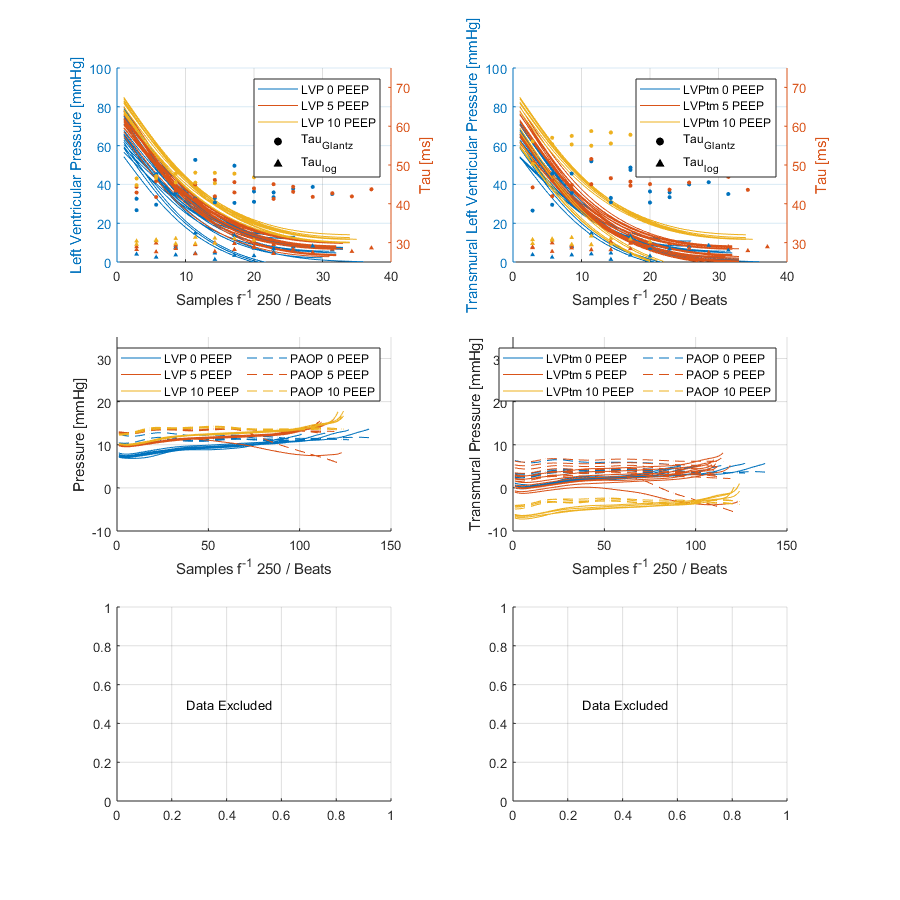


**Patient 9**


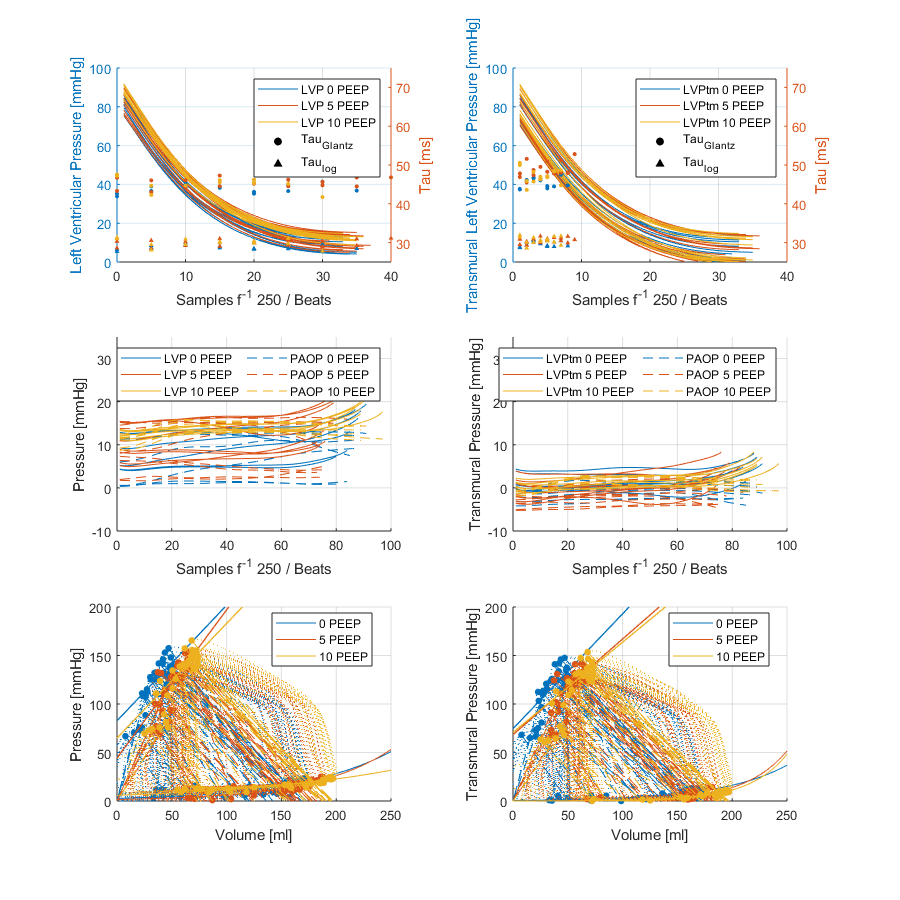


**Patient 10**


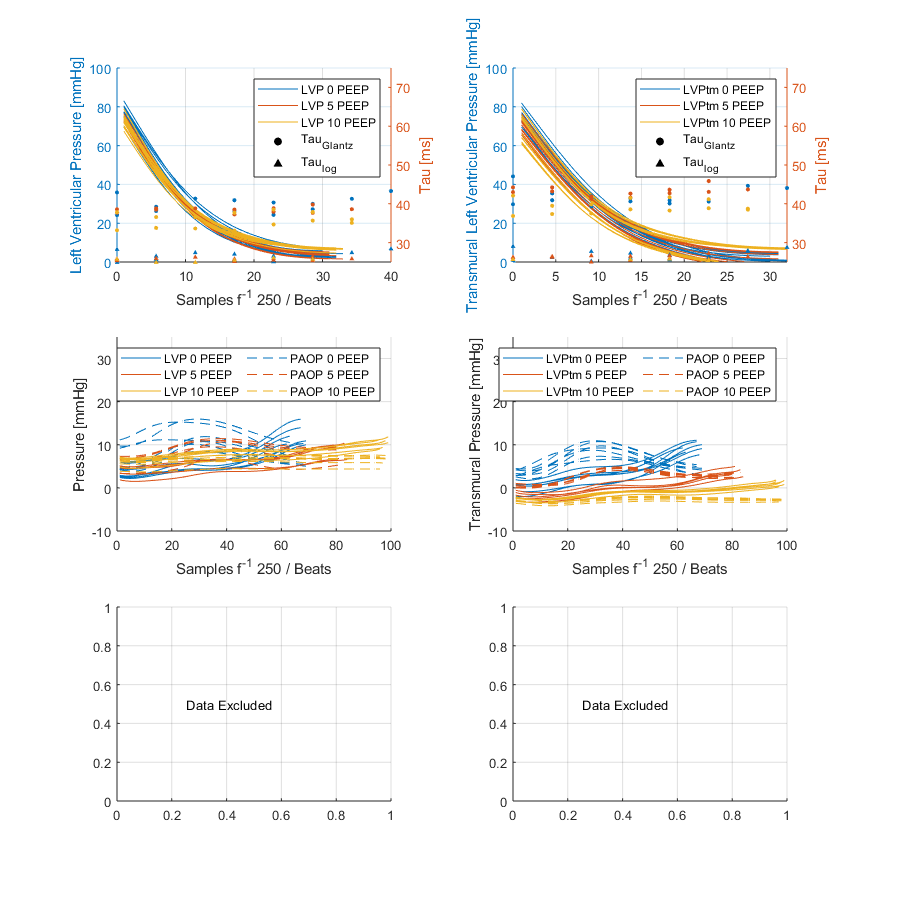


**Patient 11**


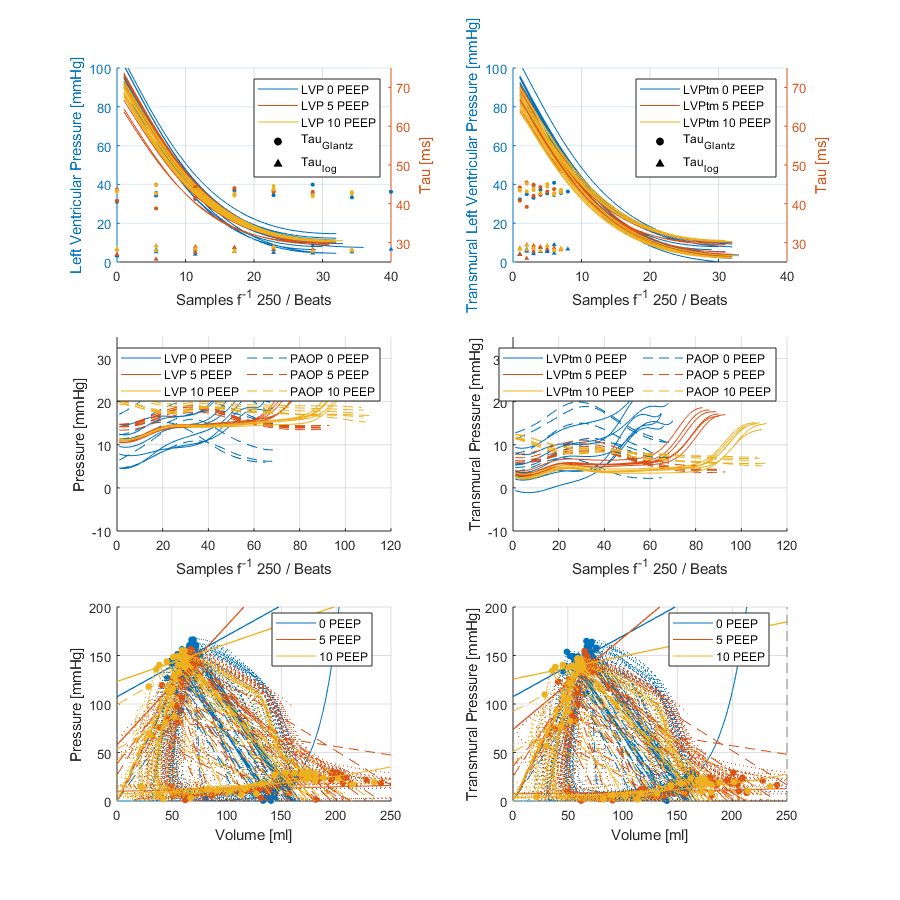


**Patient 12**


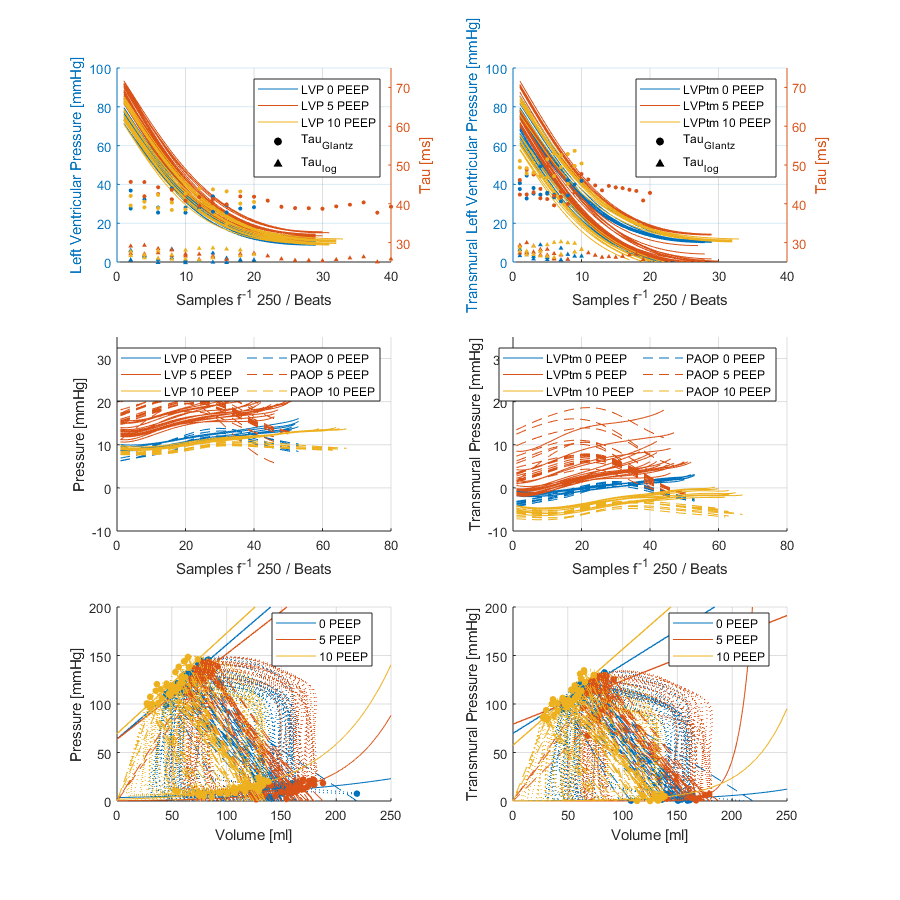


**Patient 13**


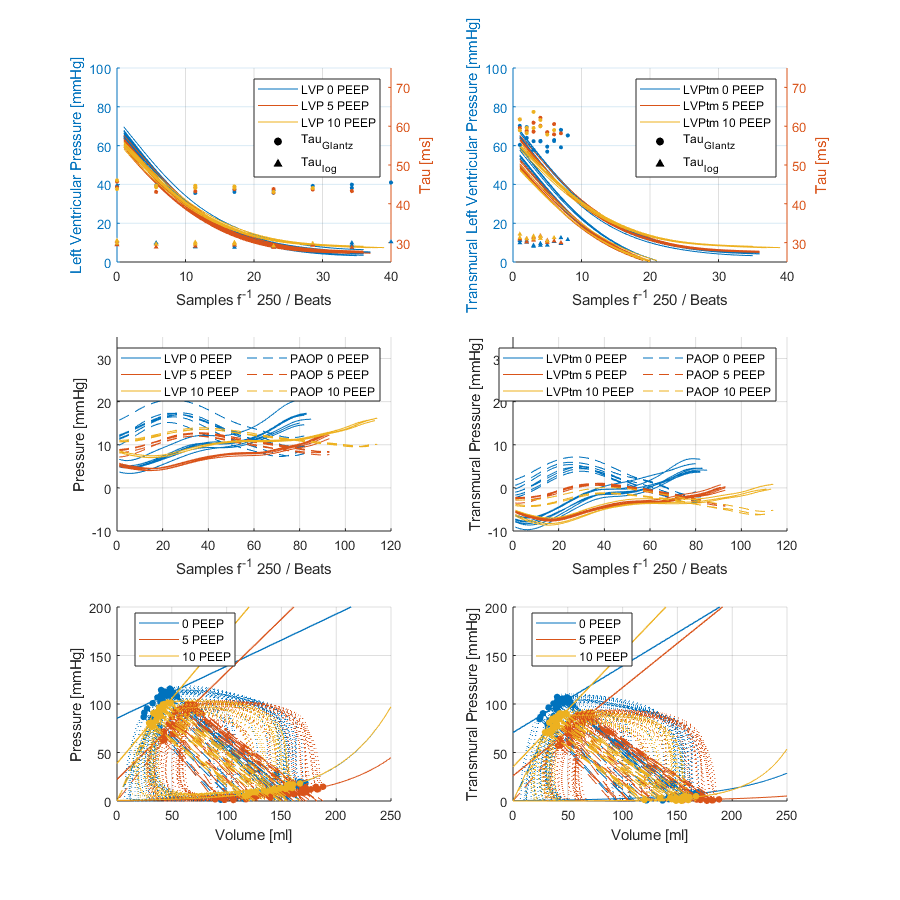


**Patient 14**


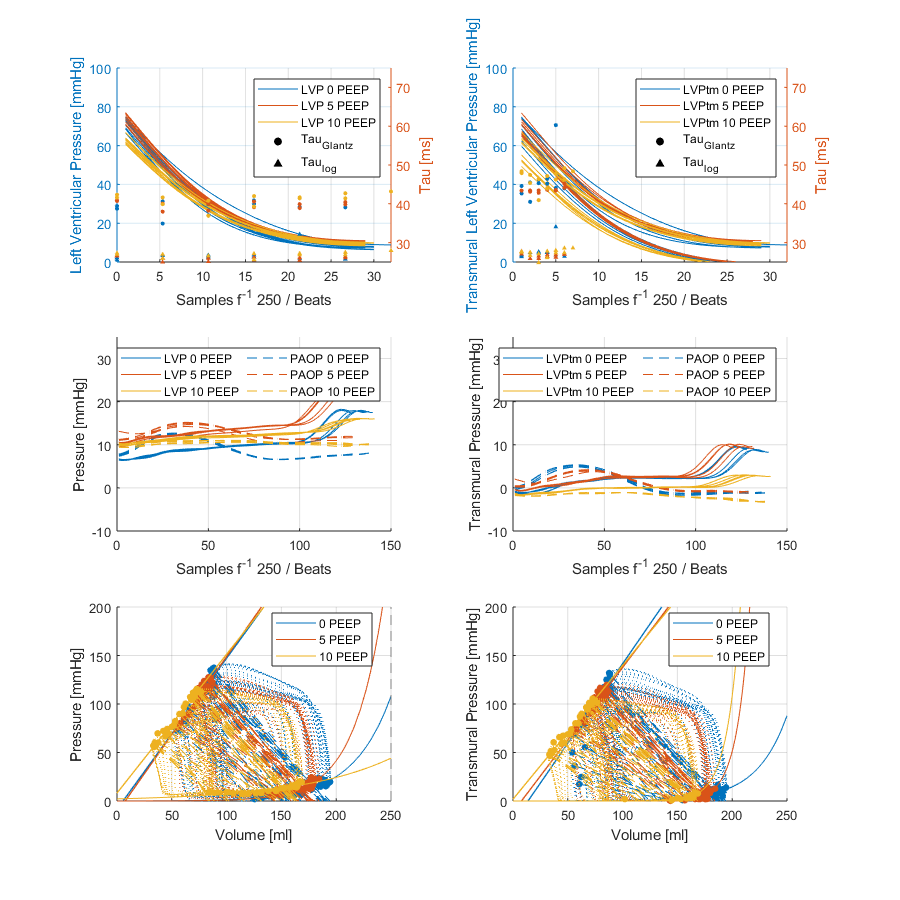


**Patient 15**


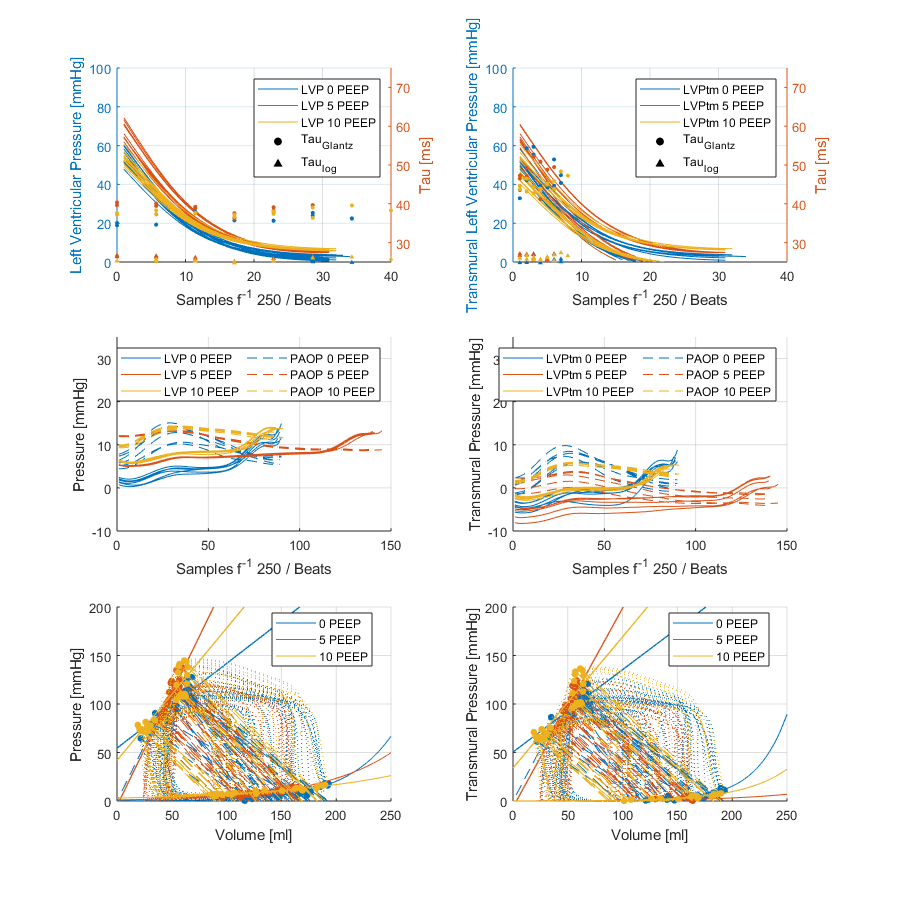


**Patient 16**


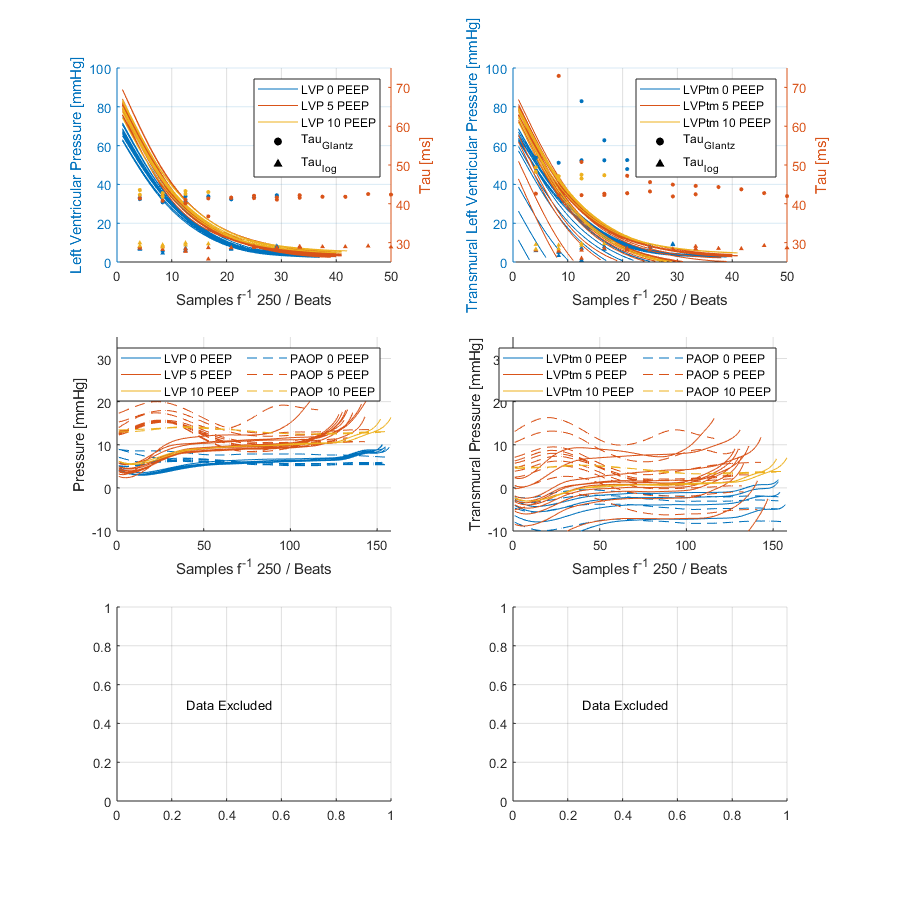


**Patient 17**


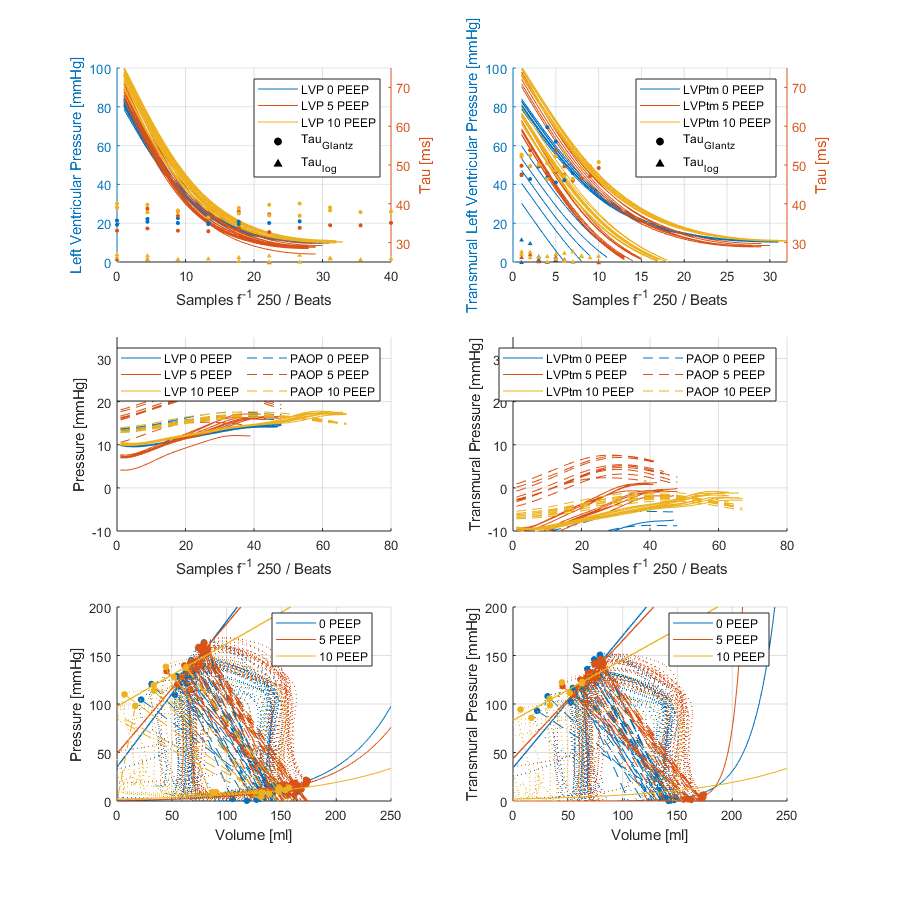


**Patient 18**


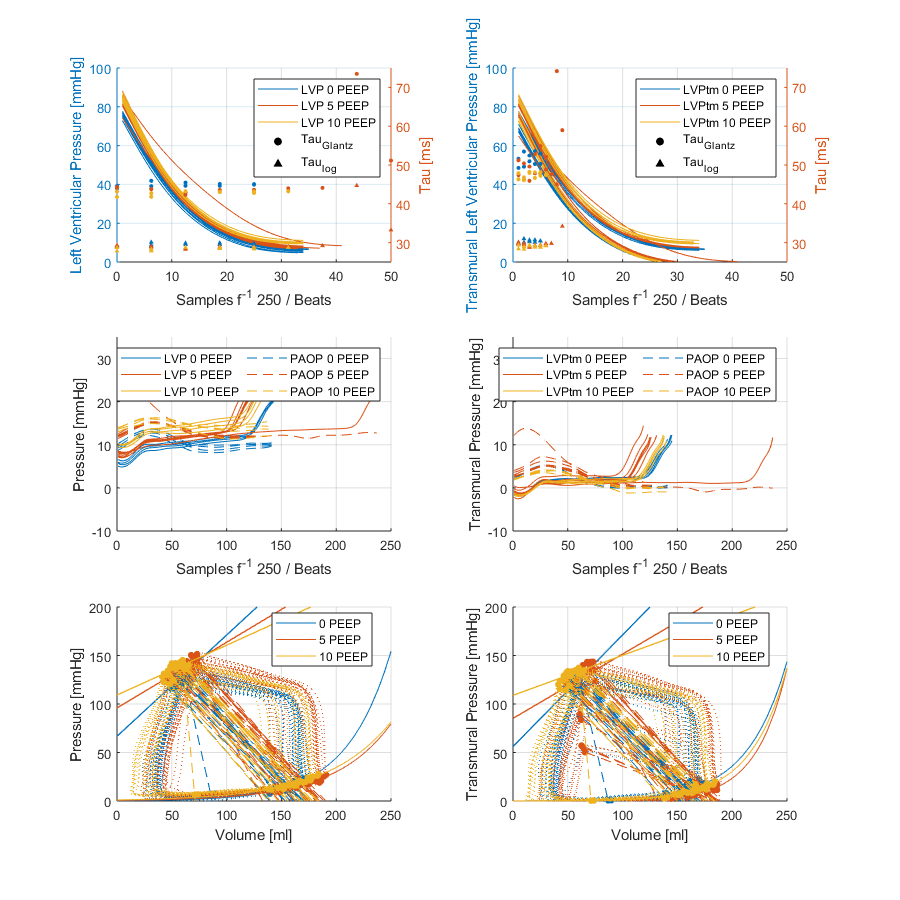


**Patient 19**


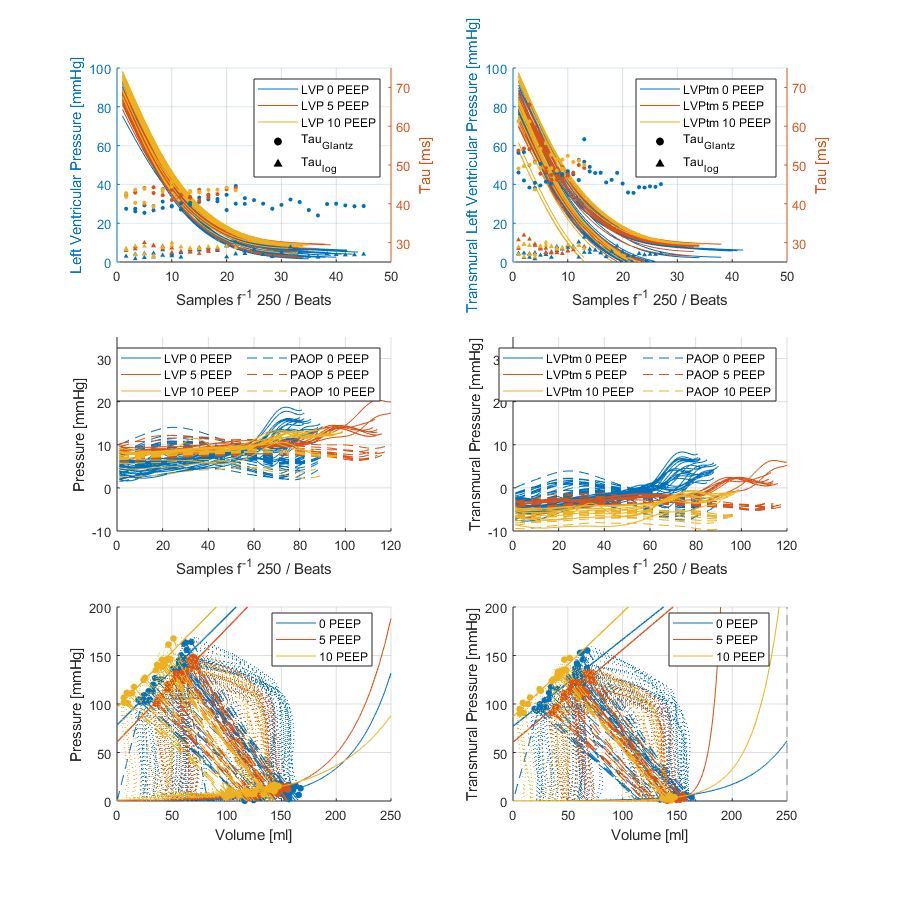


**Patient 20**


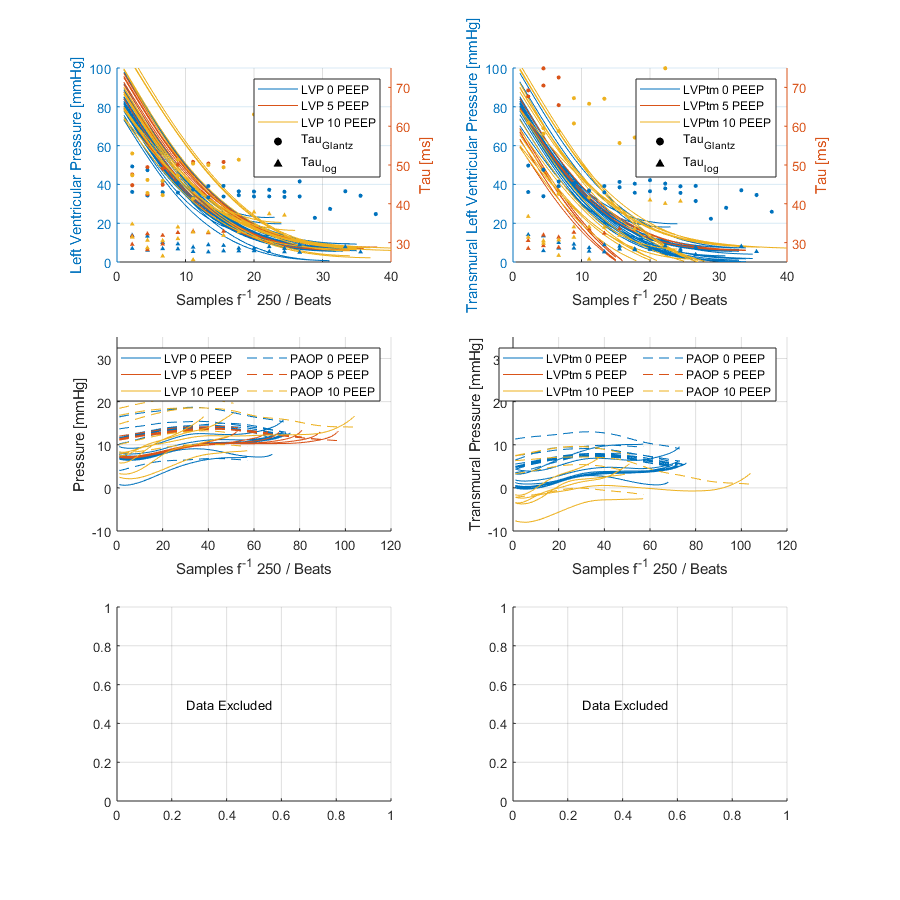


**Patient 21**


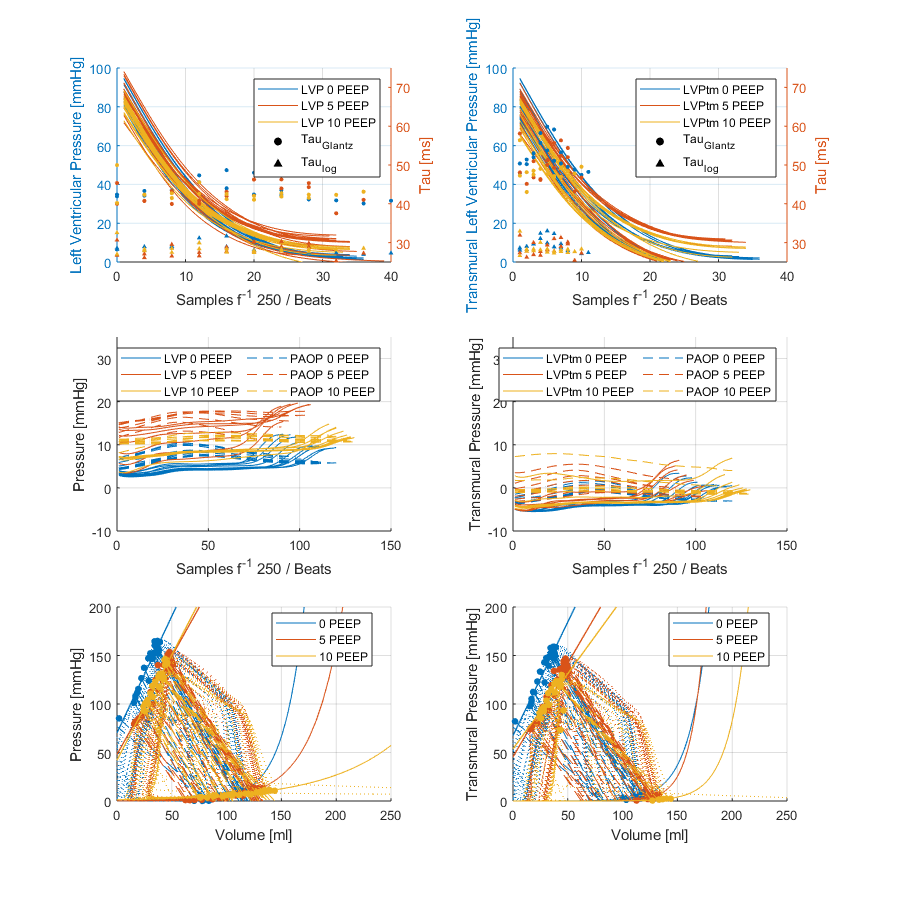


**Patient 22**


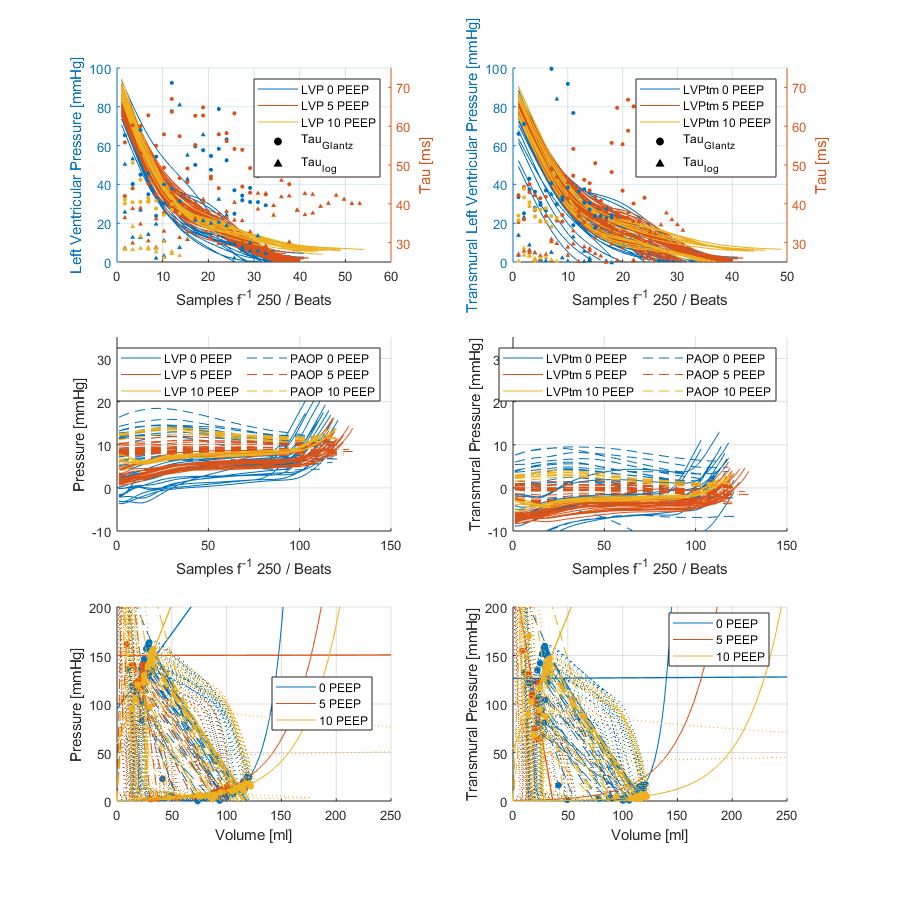


**Patient 23**


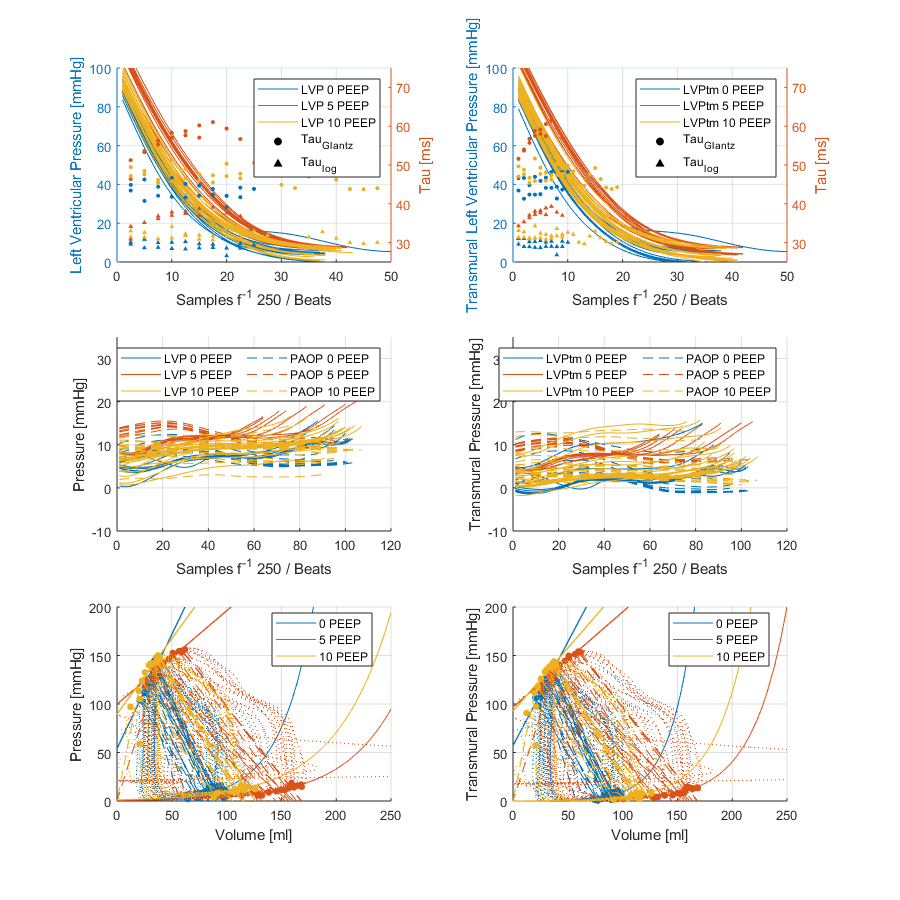


**Patient 24**


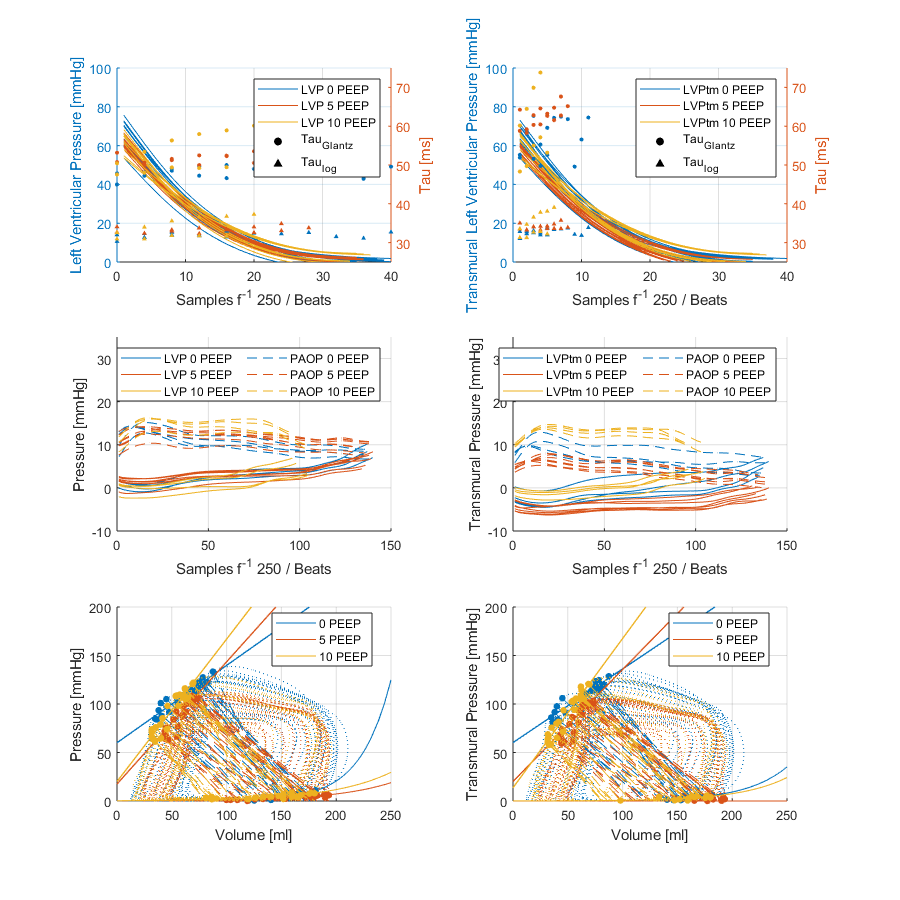


**Patient 25**


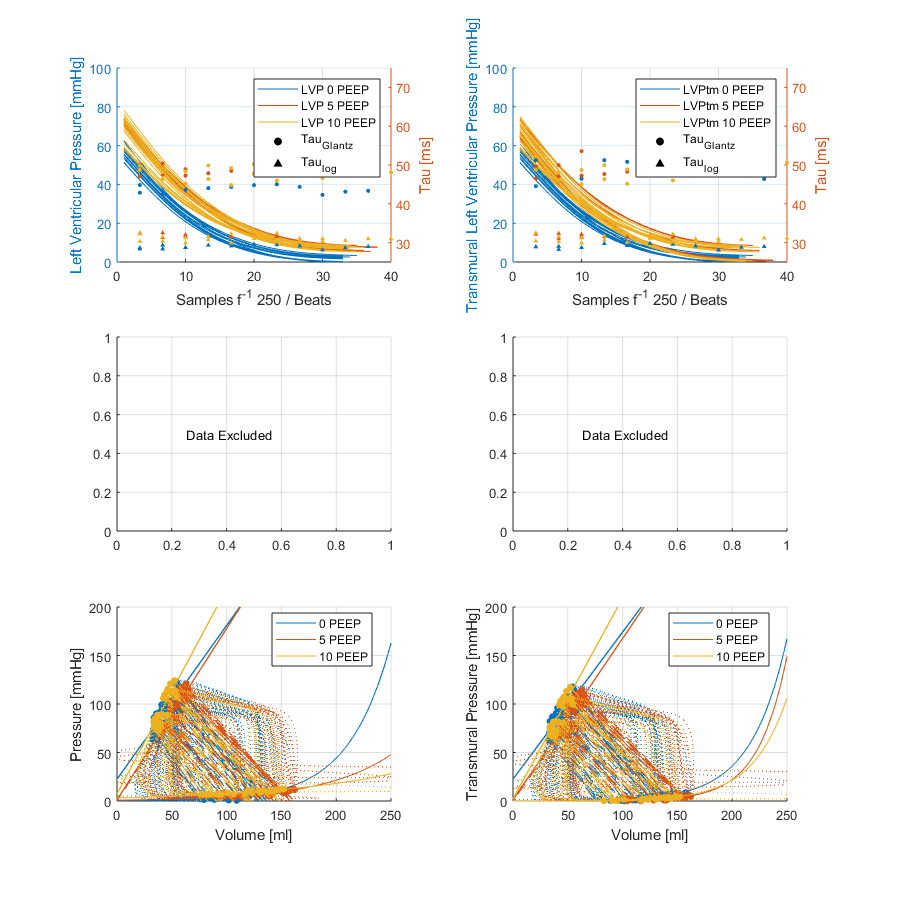


References

1. Chiumello D, Gallazzi E, Marino A, Berto V, Mietto C, Cesana B, Gattinoni L: **A validation study of a new nasogastric polyfunctional catheter**. *Intensive care medicine* 2011, **37**(5):791-795.

2. Mojoli F, Iotti GA, Torriglia F, Pozzi M, Volta CA, Bianzina S, Braschi A, Brochard L: **In vivo calibration of esophageal pressure in the mechanically ventilated patient makes measurements reliable**. *Critical care (London, England)* 2016, **20**:98.

3. Kasner M, Westermann D, Steendijk P, Drose S, Poller W, Schultheiss HP, Tschope C: **Left ventricular dysfunction induced by nonsevere idiopathic pulmonary arterial hypertension: a pressure-volume relationship study**. *Am J Respir Crit Care Med* 2012, **186**(2):181-189.

4. Kasner M, Westermann D, Steendijk P, Gaub R, Wilkenshoff U, Weitmann K, Hoffmann W, Poller W, Schultheiss HP, Pauschinger M *et al*: **Utility of Doppler echocardiography and tissue Doppler imaging in the estimation of diastolic function in heart failure with normal ejection fraction: a comparative Doppler-conductance catheterization study**. *Circulation* 2007, **116**(6):637-647.

5. LaFarge CG, Miettinen OS: **The estimation of oxygen consumption1**. *Cardiovascular research* 1970, **4**(1):23-30.

6. Ogilvie LM, Edgett BA, Huber JS, Platt MJ, Eberl HJ, Lutchmedial S, Brunt KR, Simpson JA: **Hemodynamic assessment of diastolic function for experimental models**. *American journal of physiology Heart and circulatory physiology* 2020, **318**(5):H1139-h1158.
