# Supplementary material for: The effects of positive end-expiratory pressure on cardiac function: a comparative echocardiography-conductance catheter study
Source: Clin Res Cardiol. 2022 Apr 6;111(6):705–19. doi: 10.1007/s00392-022-02014-1 (PMC9151717; doi:10.1007/s00392-022-02014-1)

Individual patient data

**Patient 1**
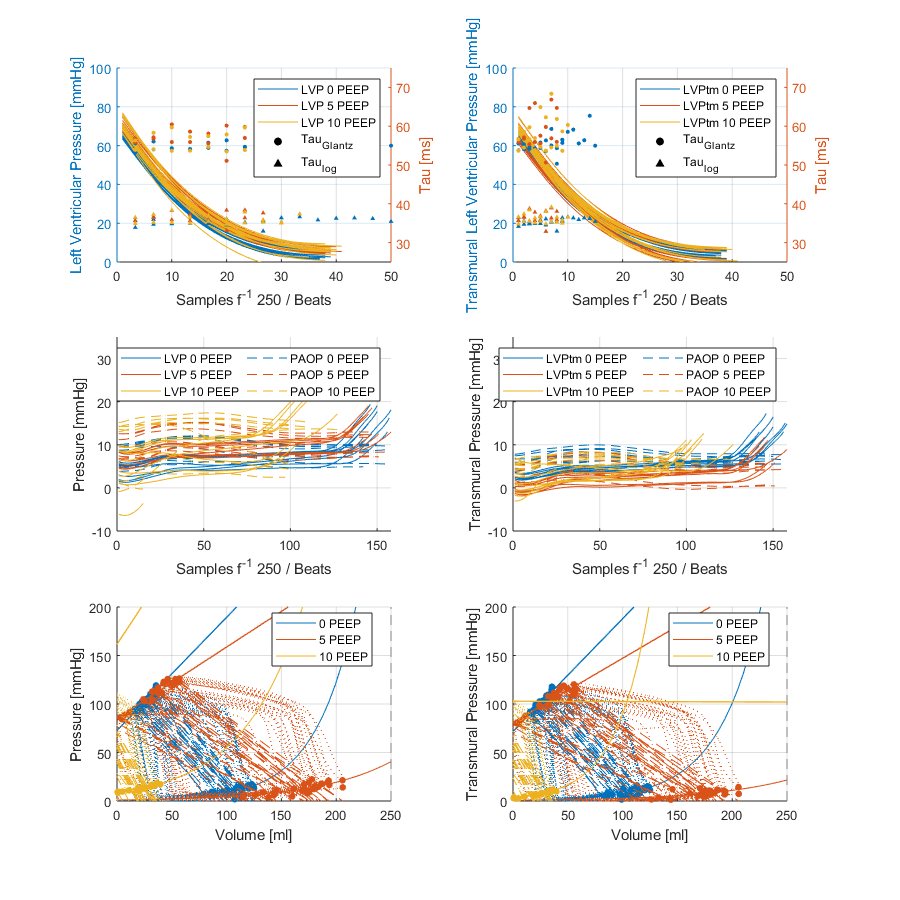


**Patient 2**


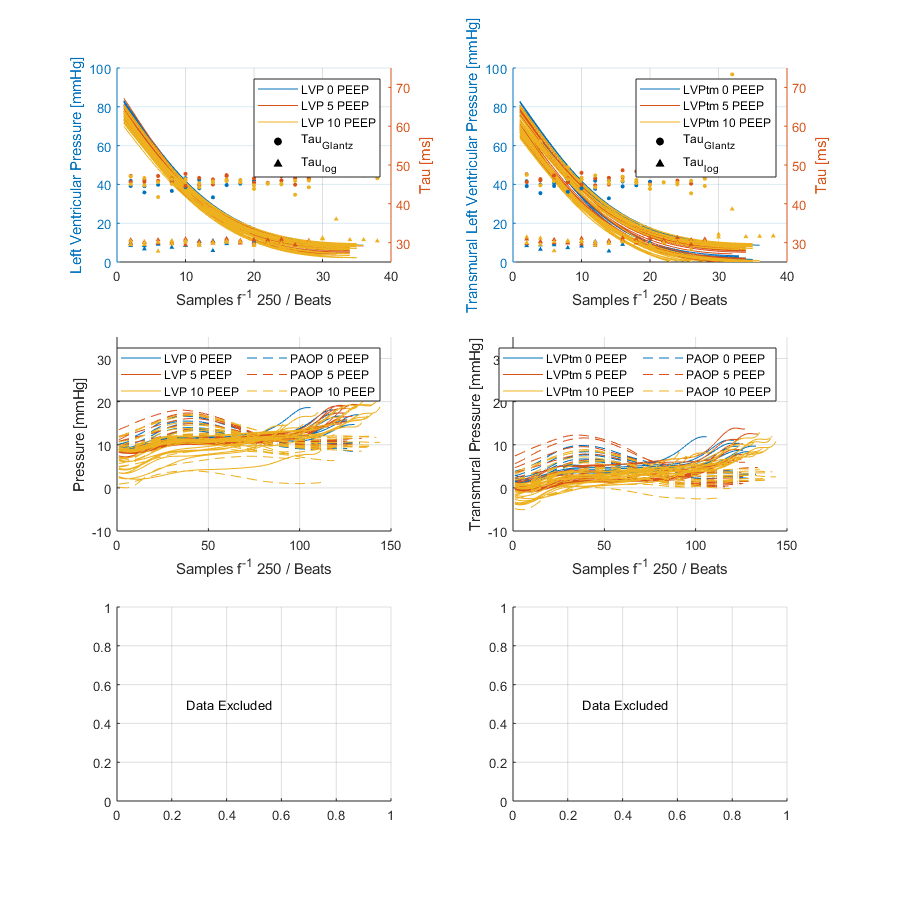


**Patient 3**


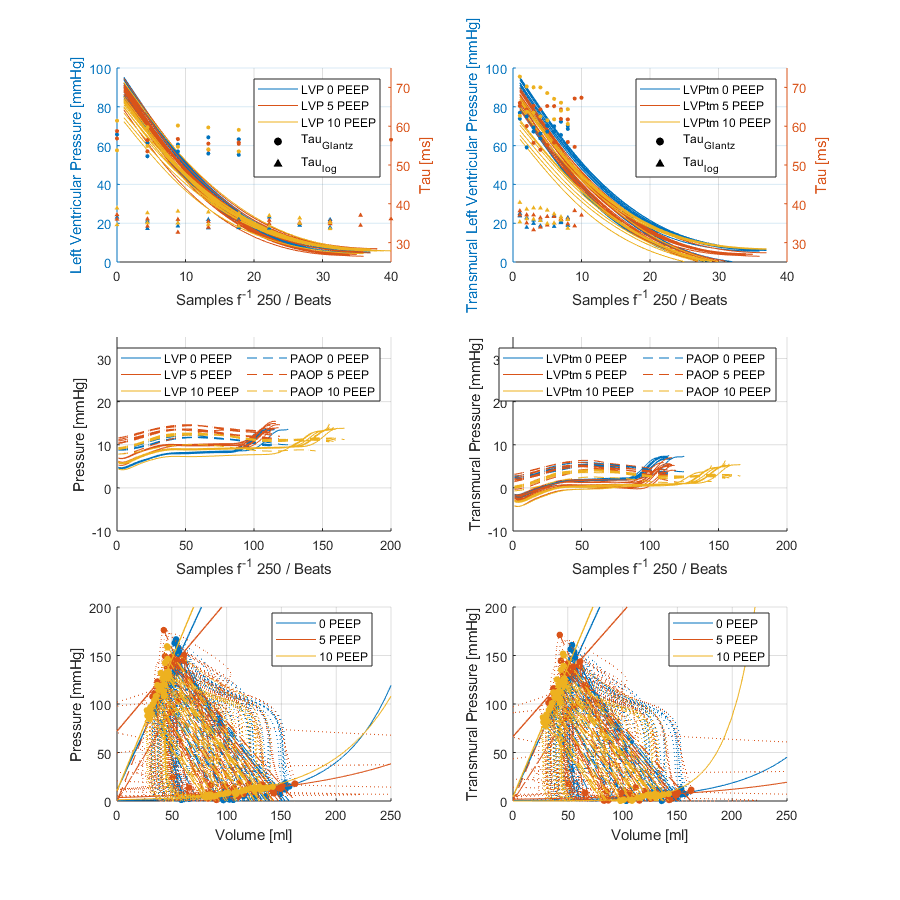


**Patient 4**


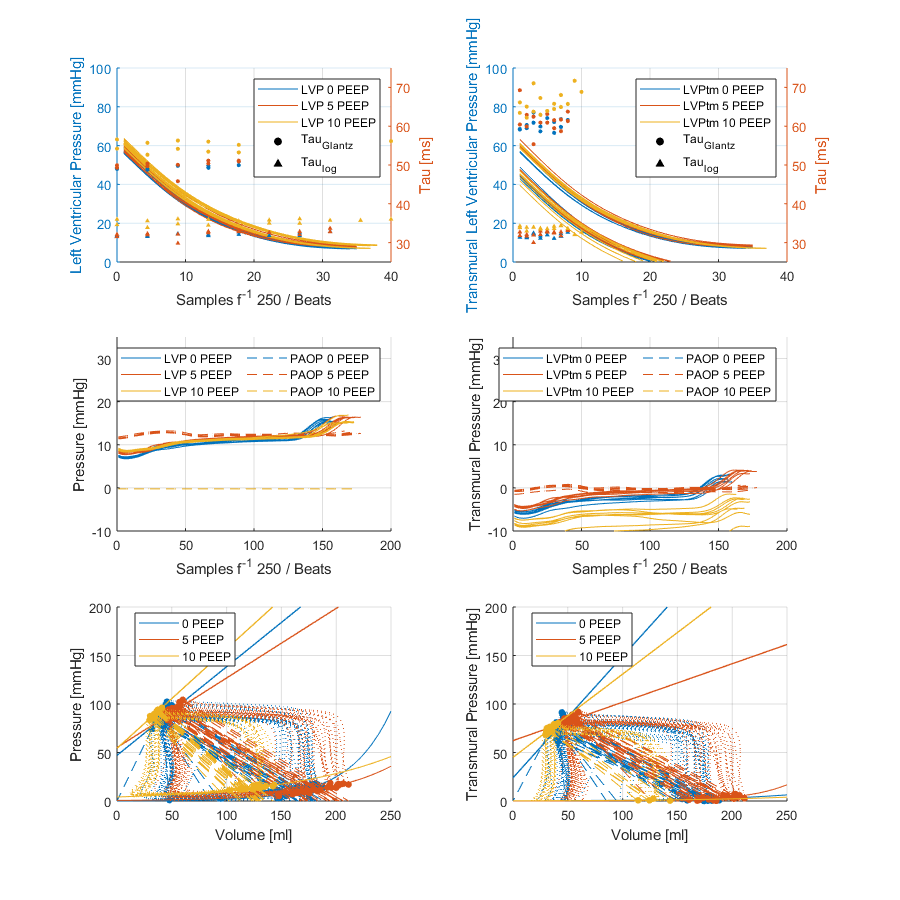


**Patient 5**


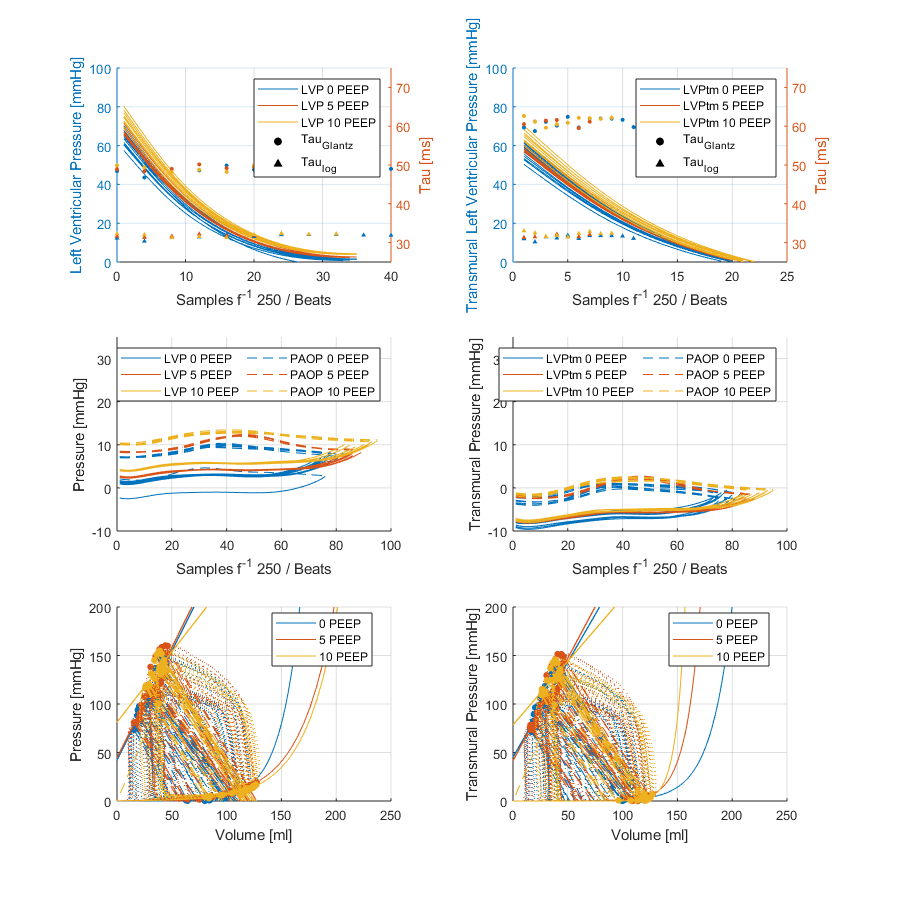


**Patient 6**


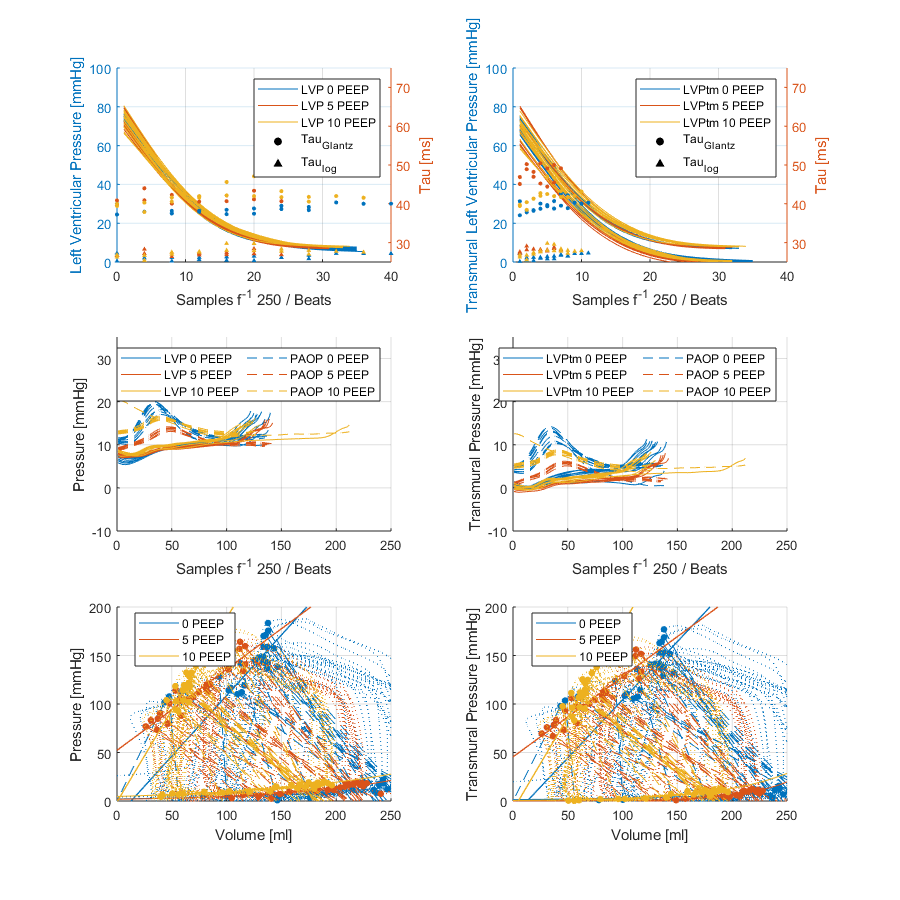


**Patient 7**


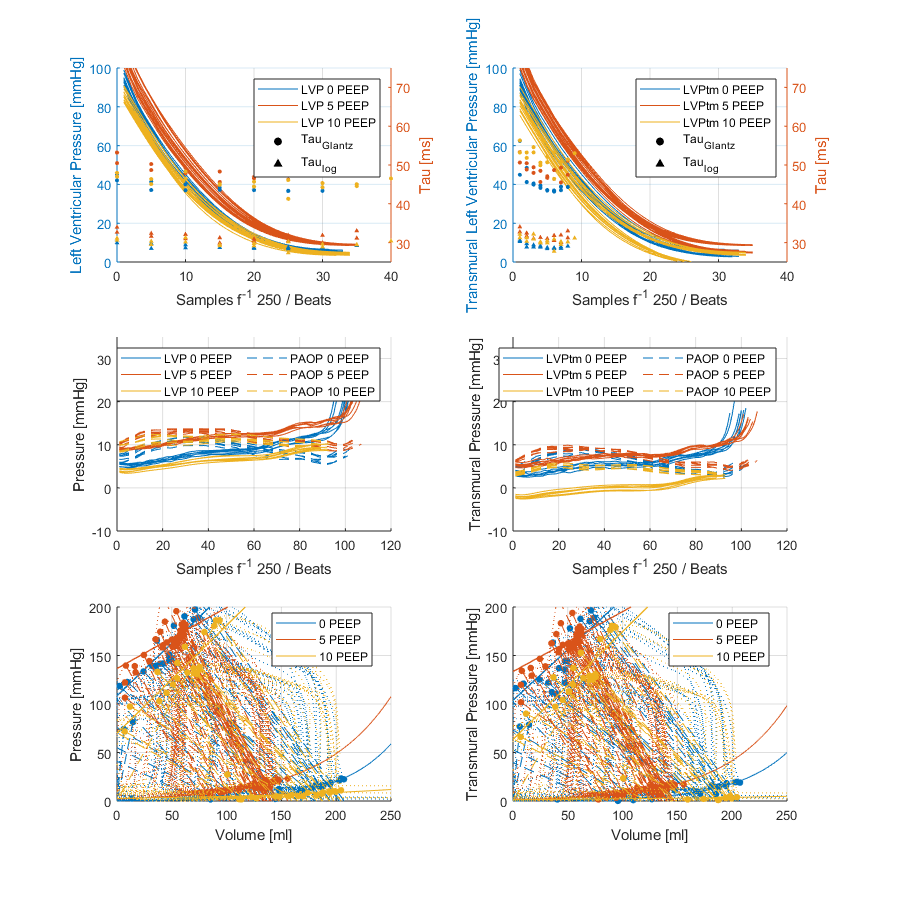


**Patient 8**


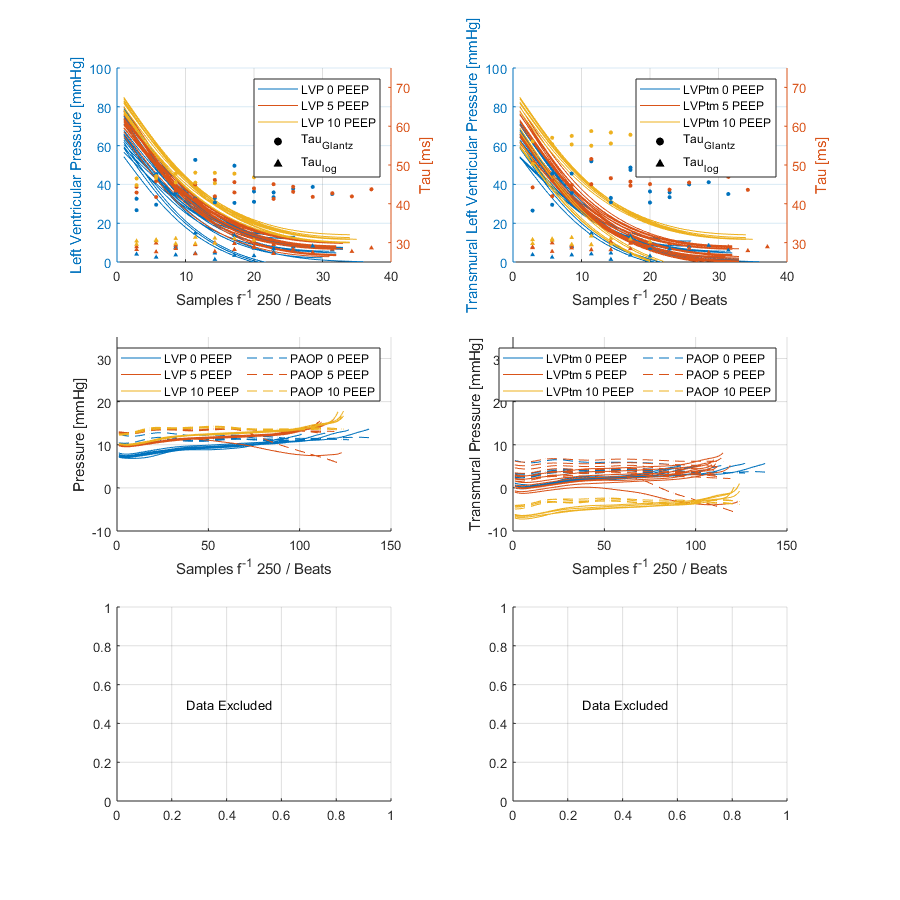


**Patient 9**


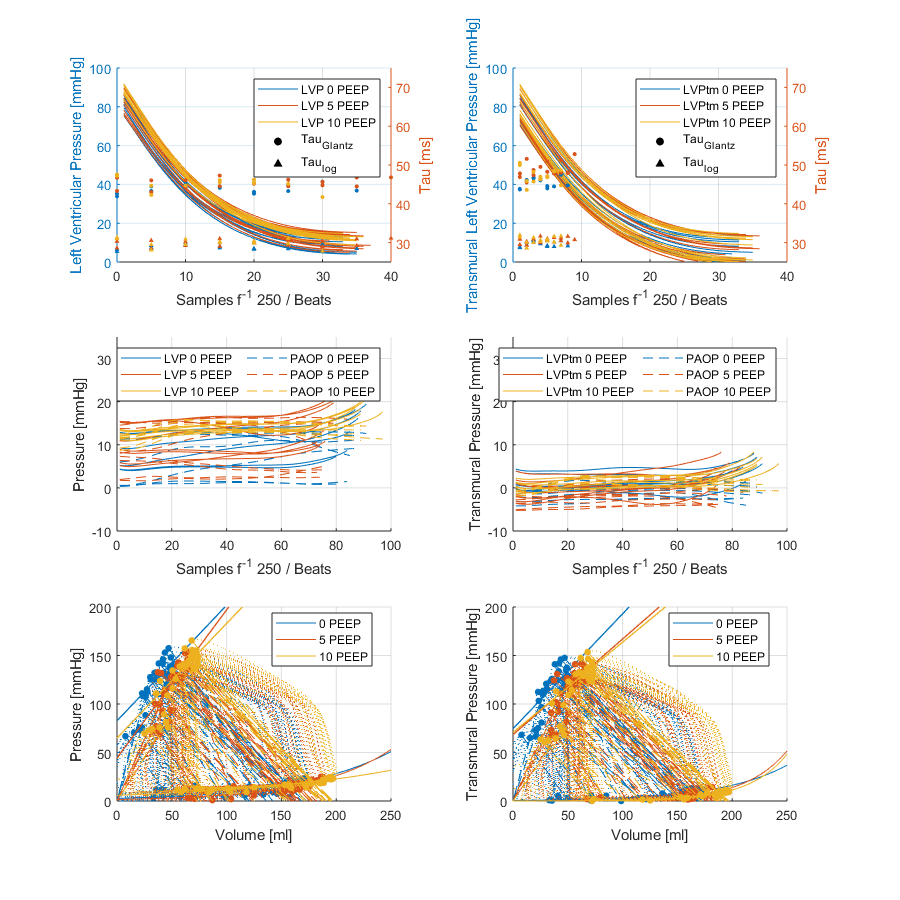


**Patient 10**


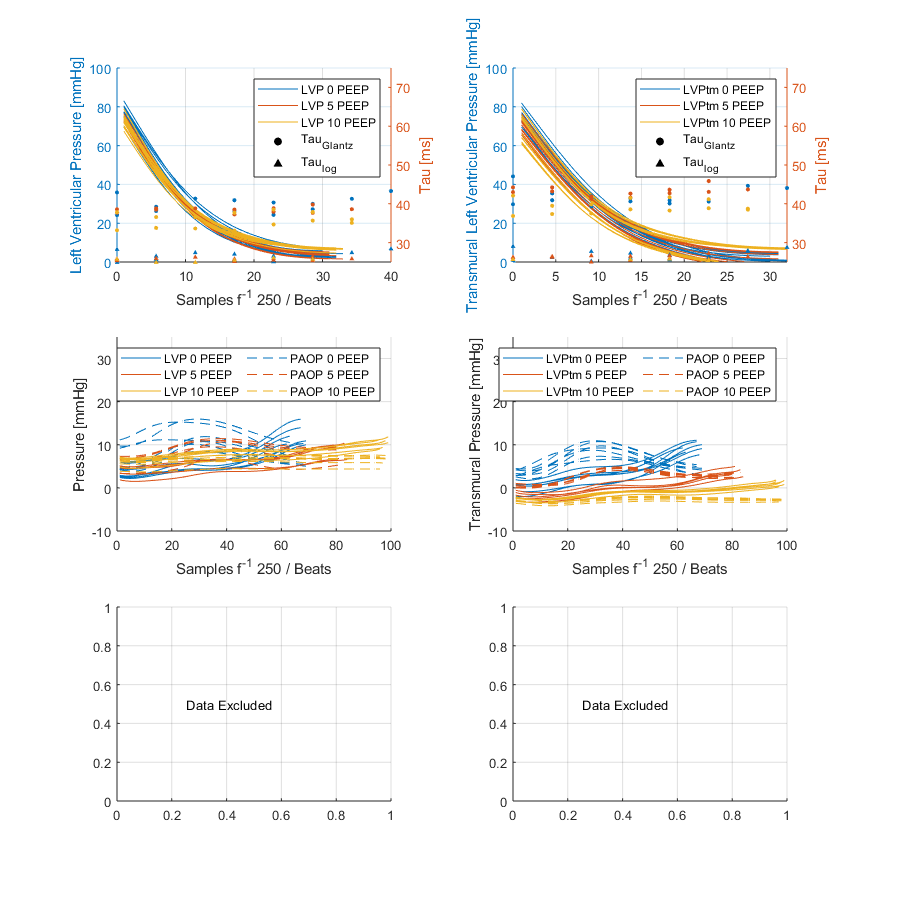


**Patient 11**


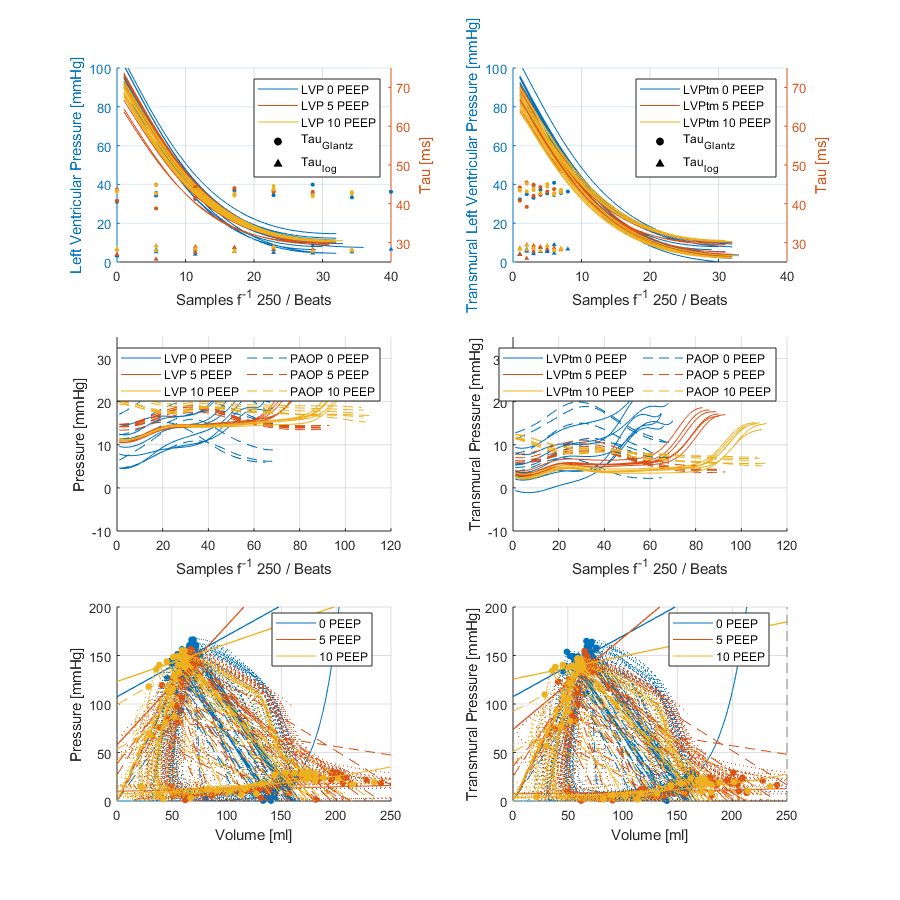


**Patient 12**


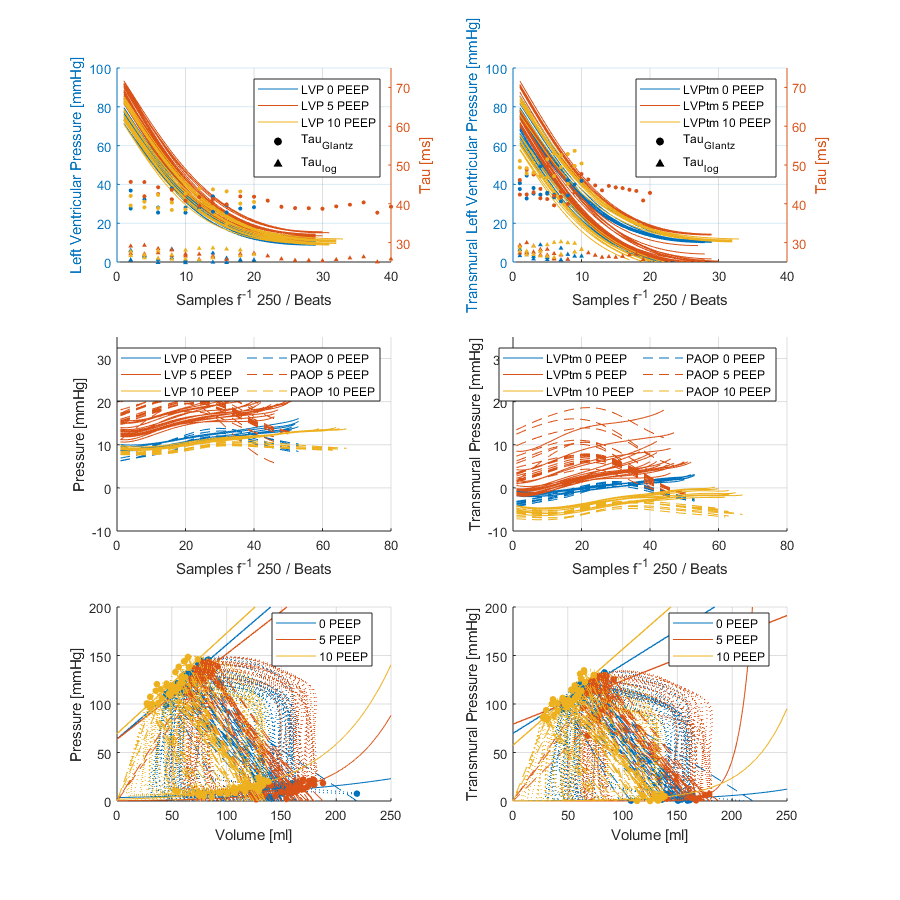


**Patient 13**


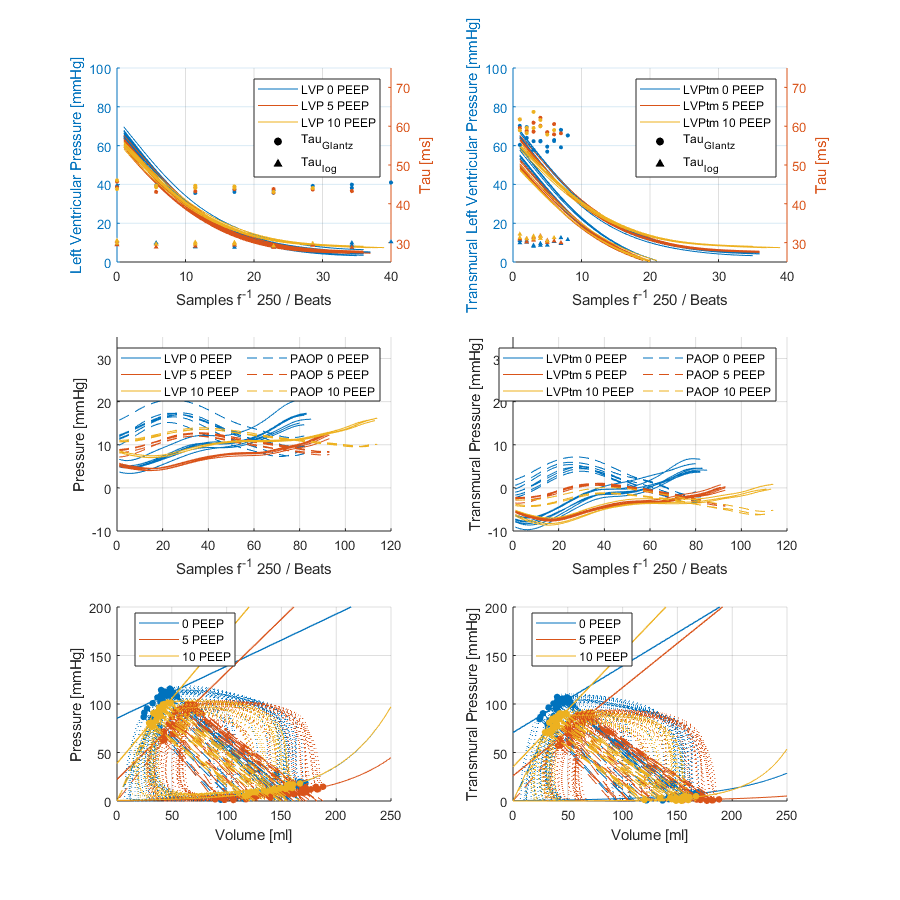


**Patient 14**


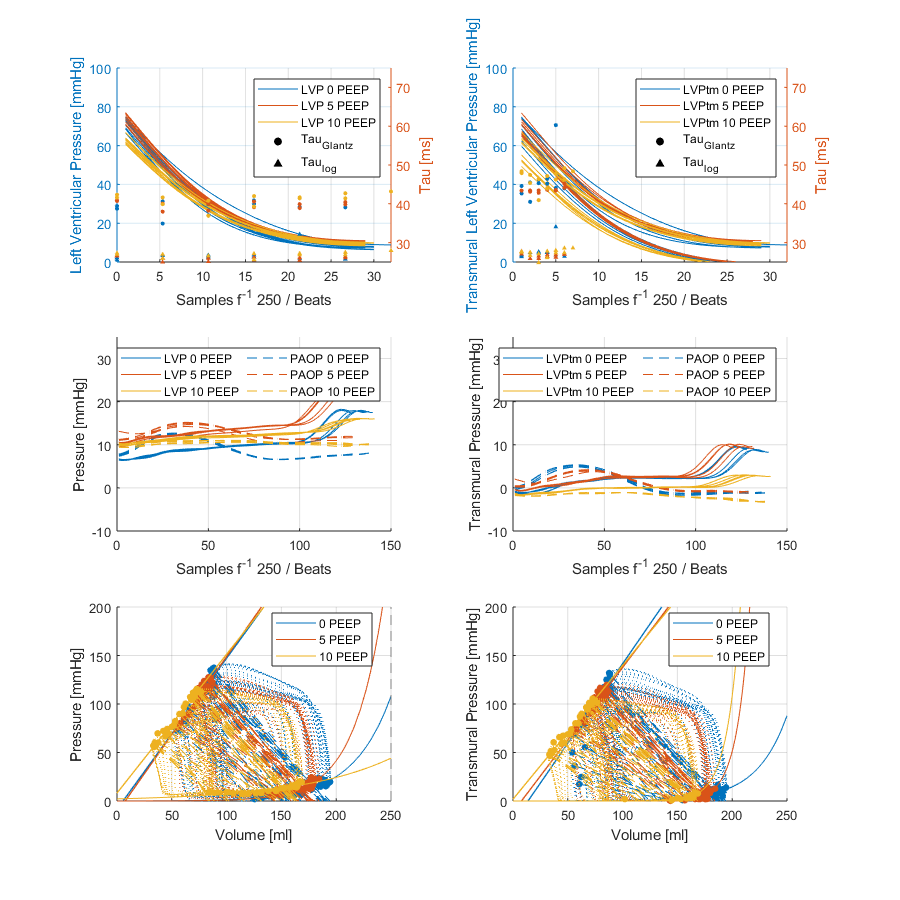


**Patient 15**


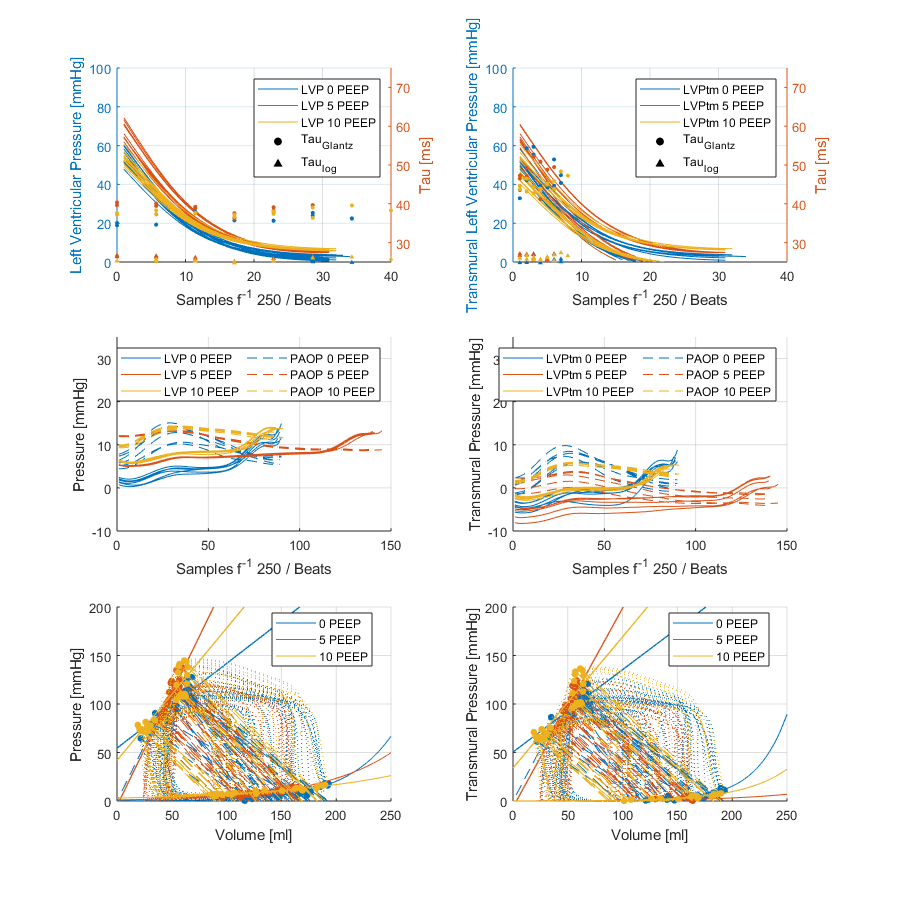


**Patient 16**


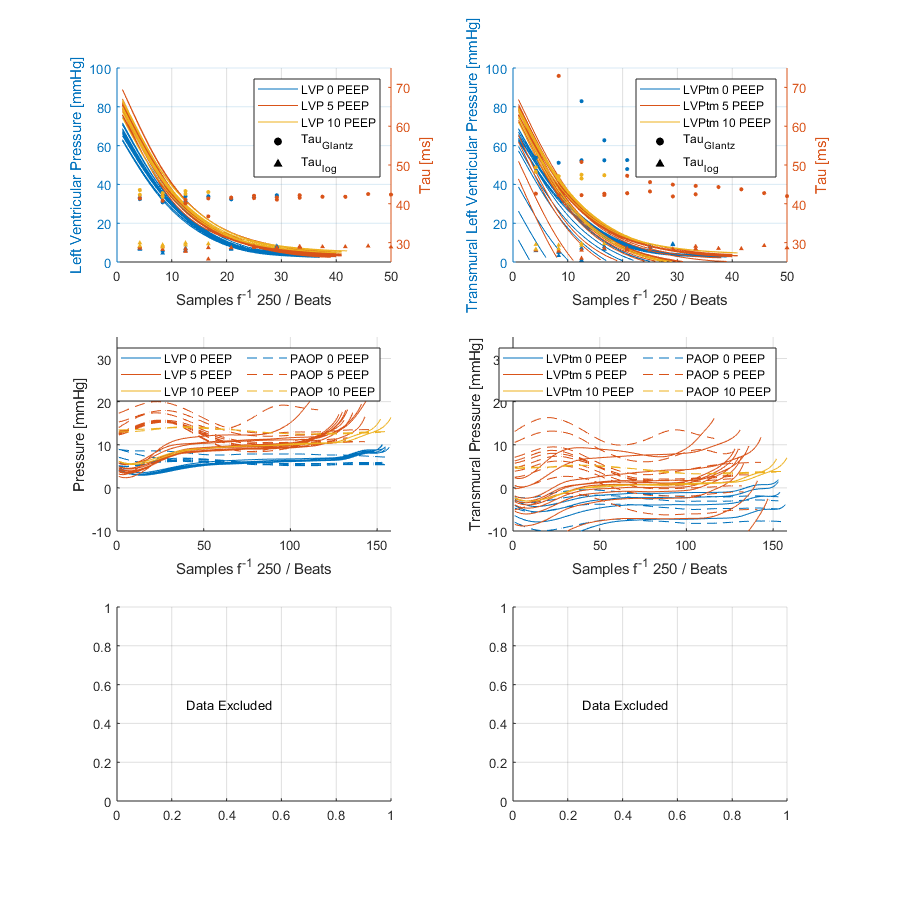


**Patient 17**


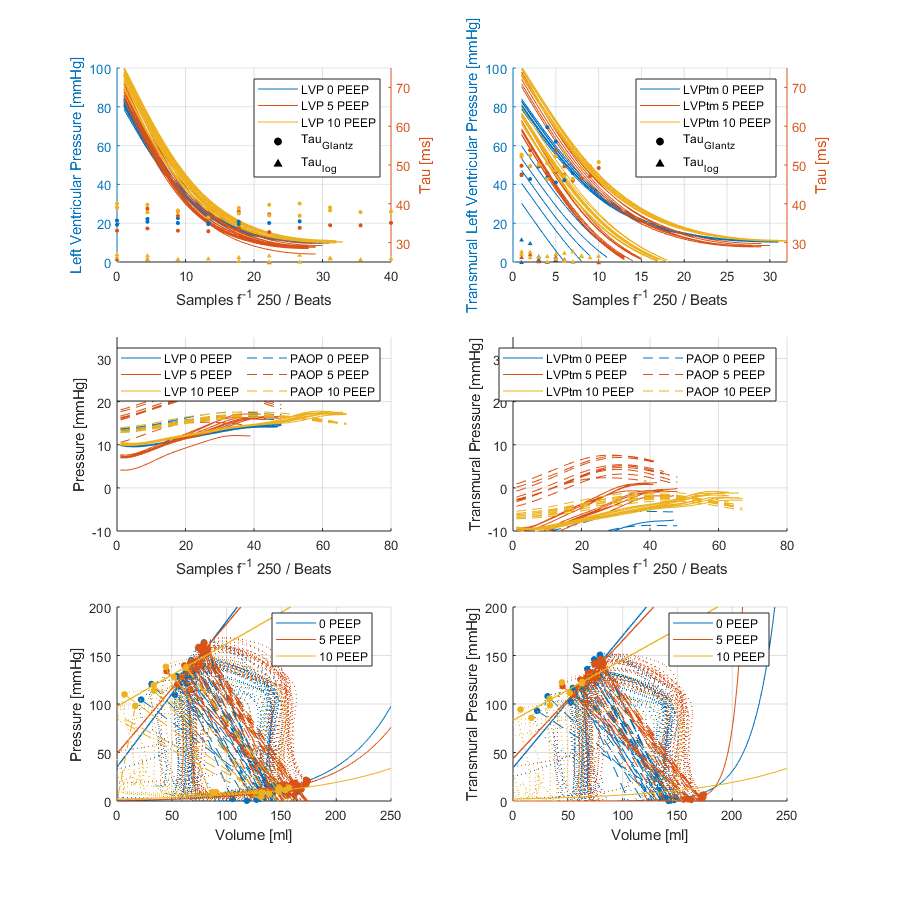


**Patient 18**


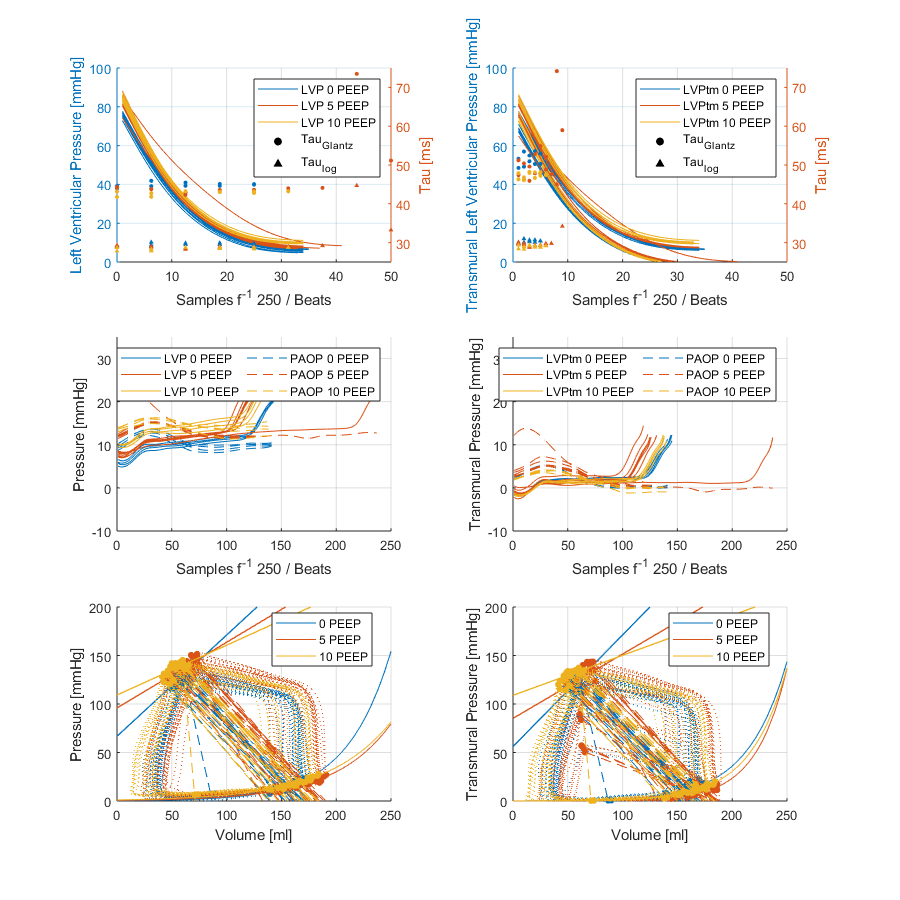


**Patient 19**


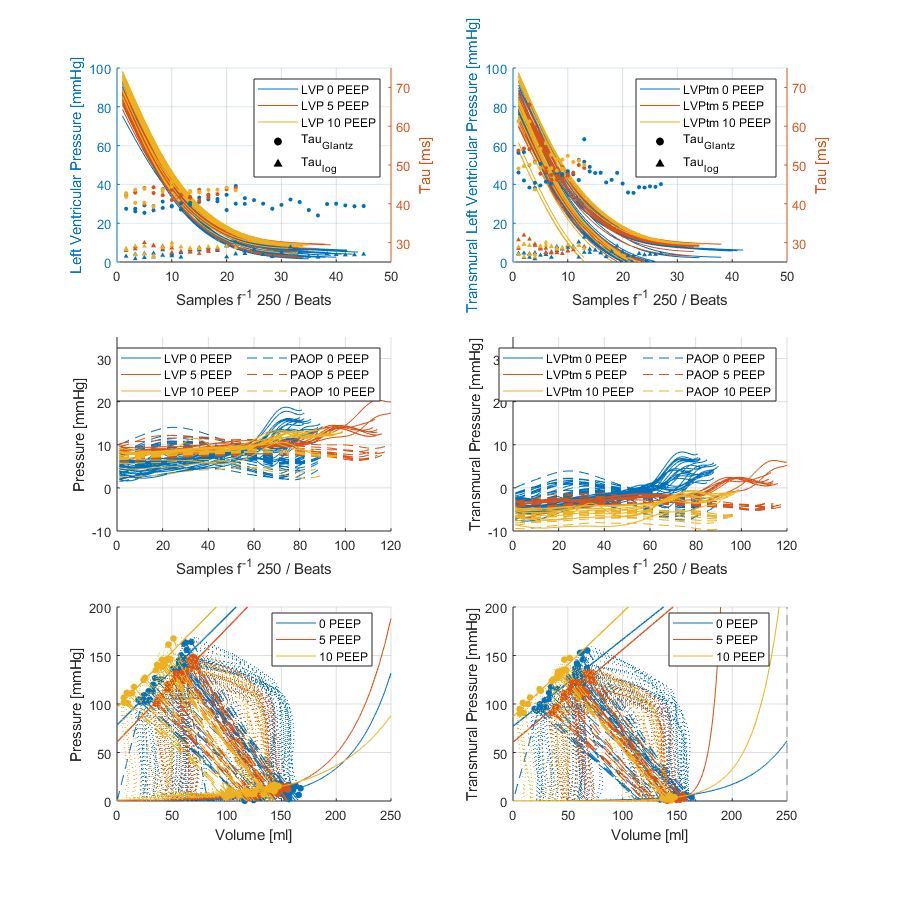


**Patient 20**


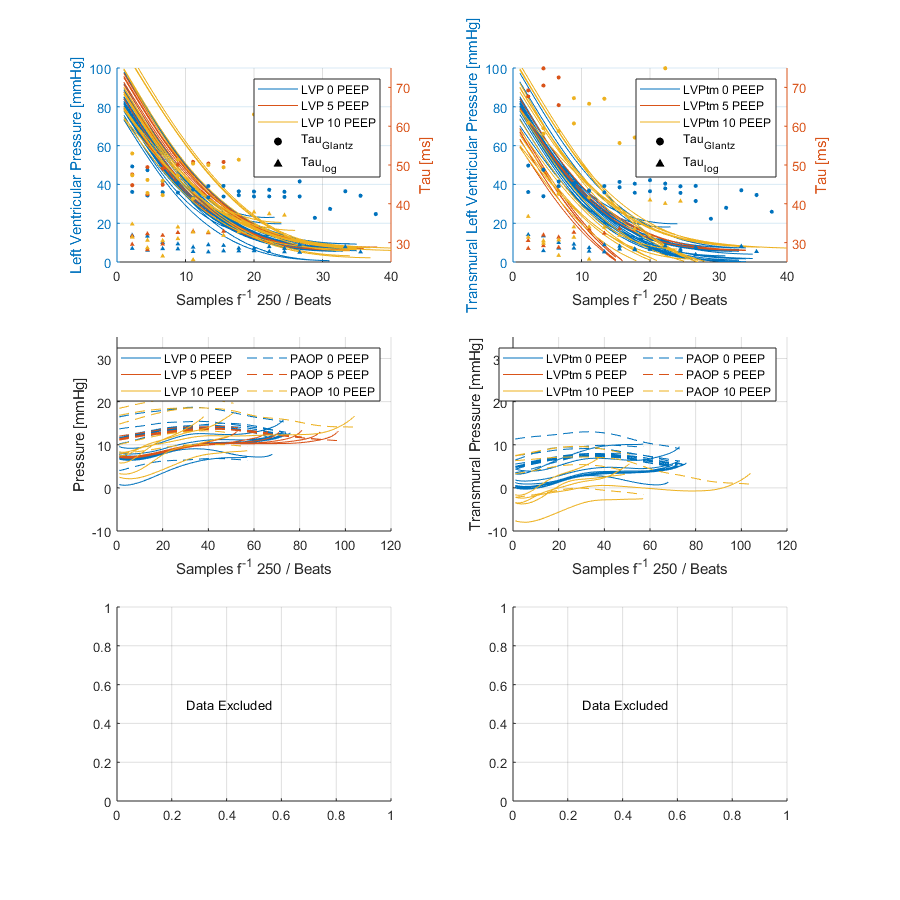


**Patient 21**


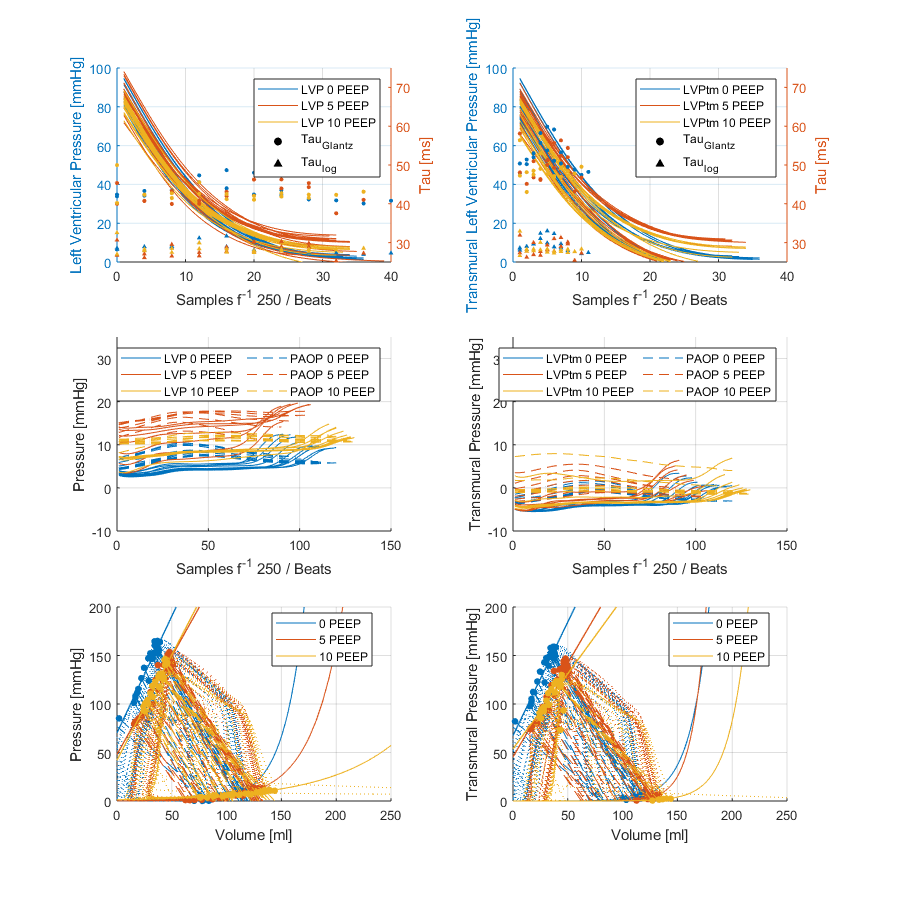


**Patient 22**


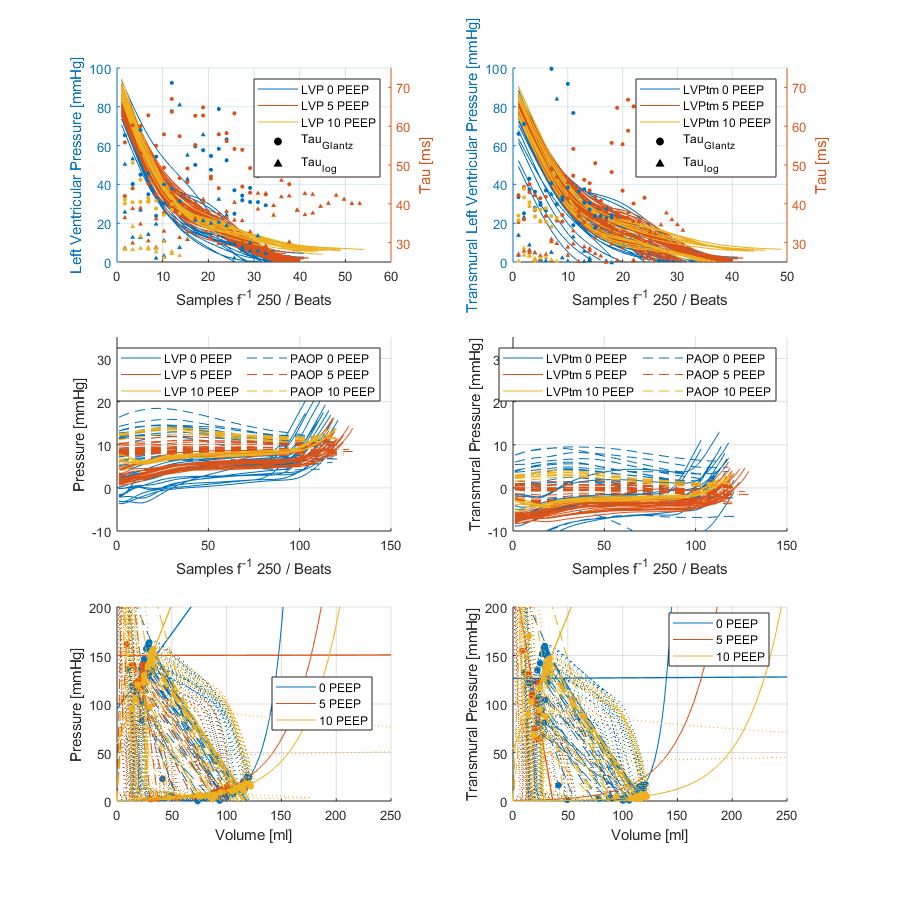


**Patient 23**


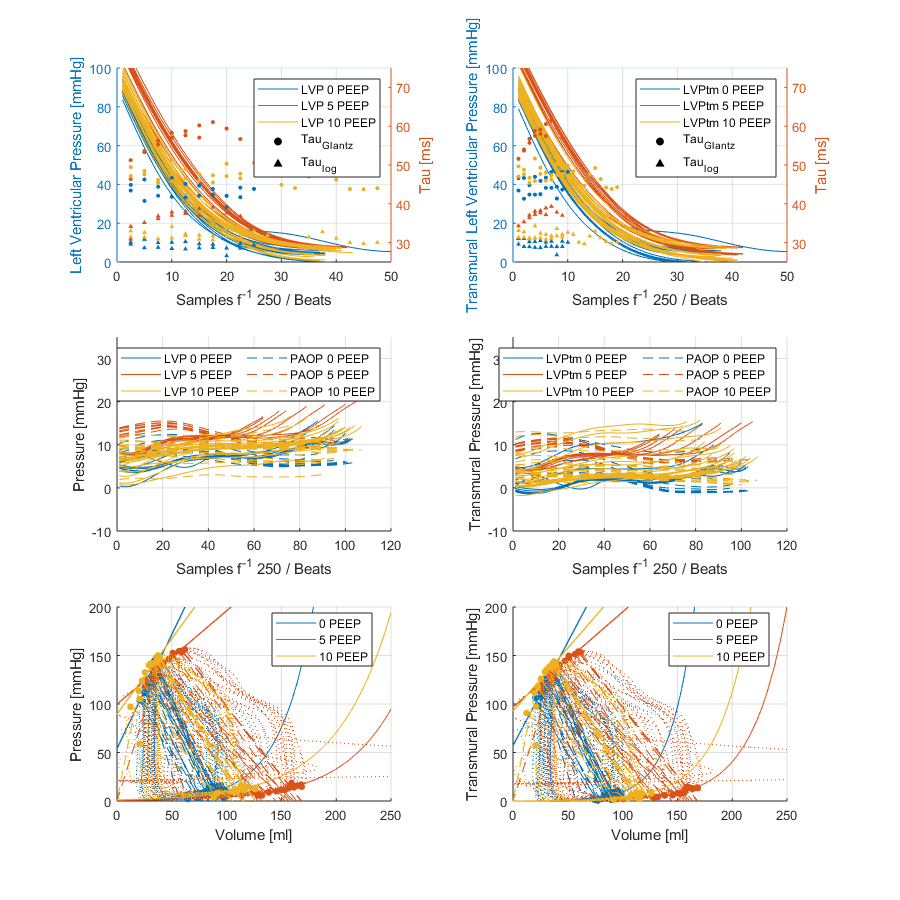


**Patient 24**


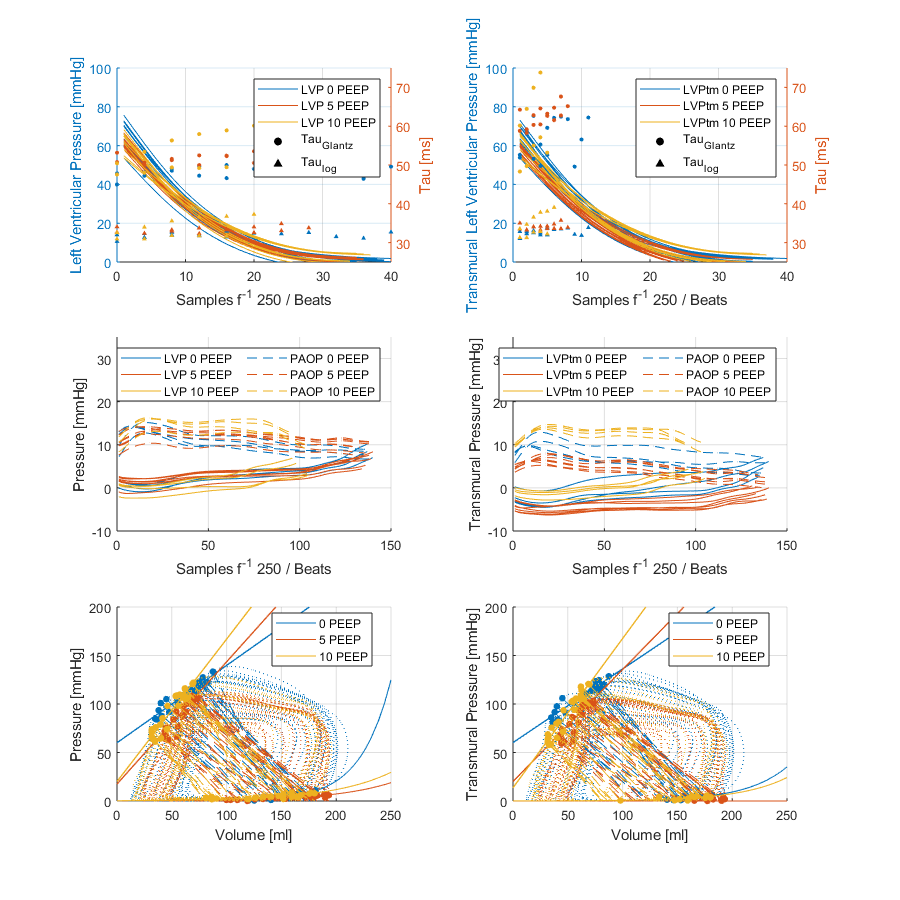


**Patient 25**


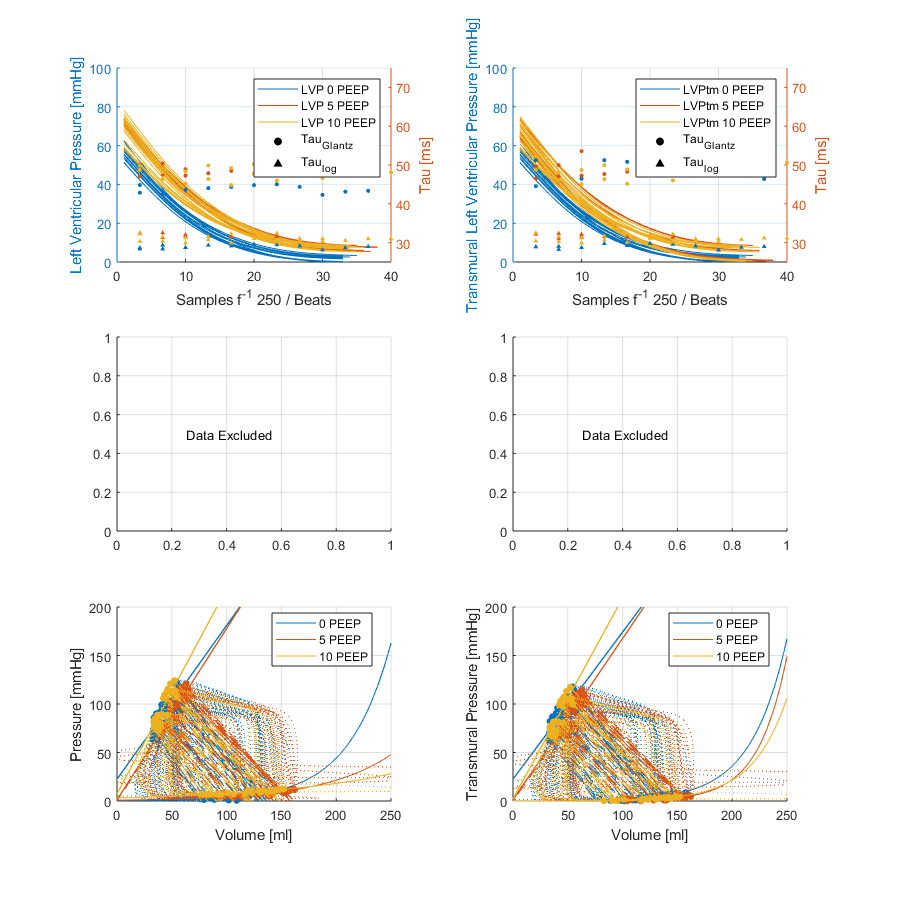

Supplement: Supplementary file 2 — Supplementary file2 (DOCX 8659 kb) [file 392_2022_2014_MOESM2_ESM.docx]
